# Supplementary material for: The role of activated androgen receptor in cofilin phospho-regulation depends on the molecular subtype of TNBC cell line and actin assembly dynamics
Source: PLoS One. 2022 Dec 30;17(12):e0279746. doi: 10.1371/journal.pone.0279746 (PMC9803305; doi:10.1371/journal.pone.0279746)
Supplement: S1 Raw images — (PDF) [file pone.0279746.s002.pdf]

Figure 1A

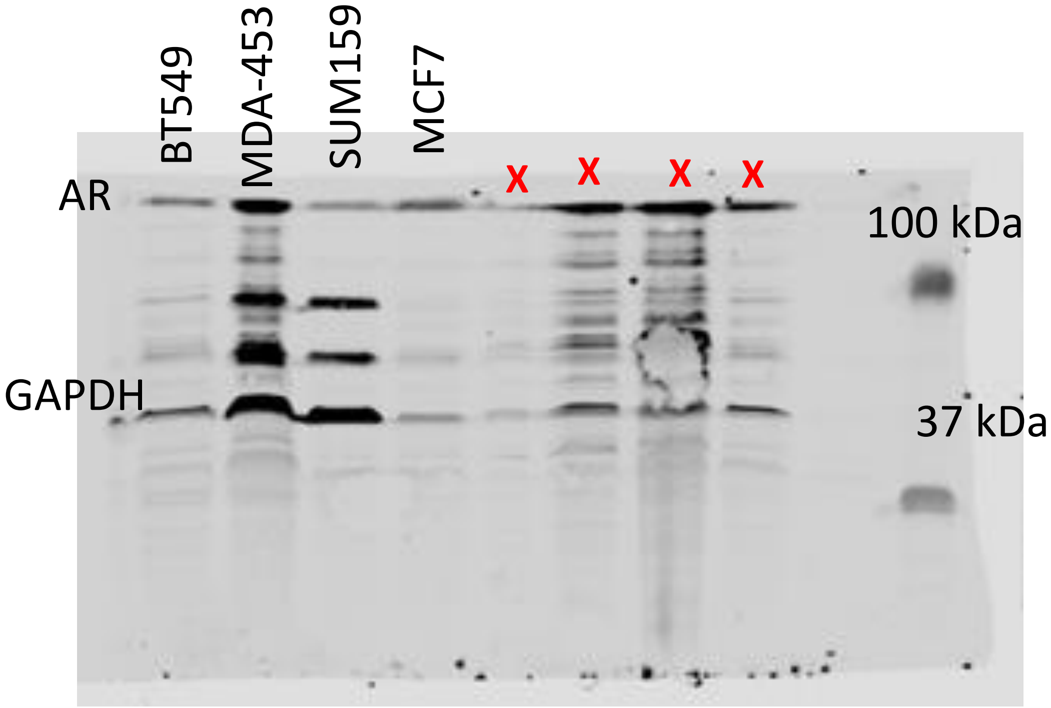
BT549
MDA-453
SUM159
MCF7
X
X
X
X
AR
GAPDH
100 kDa
37 kDa

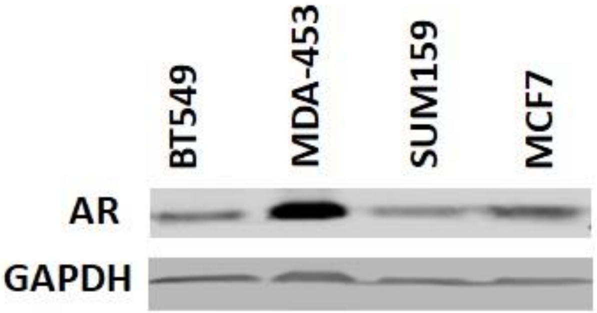
BT549
MDA-453
SUM159
MCF7
AR
GAPDH

Figure 1C

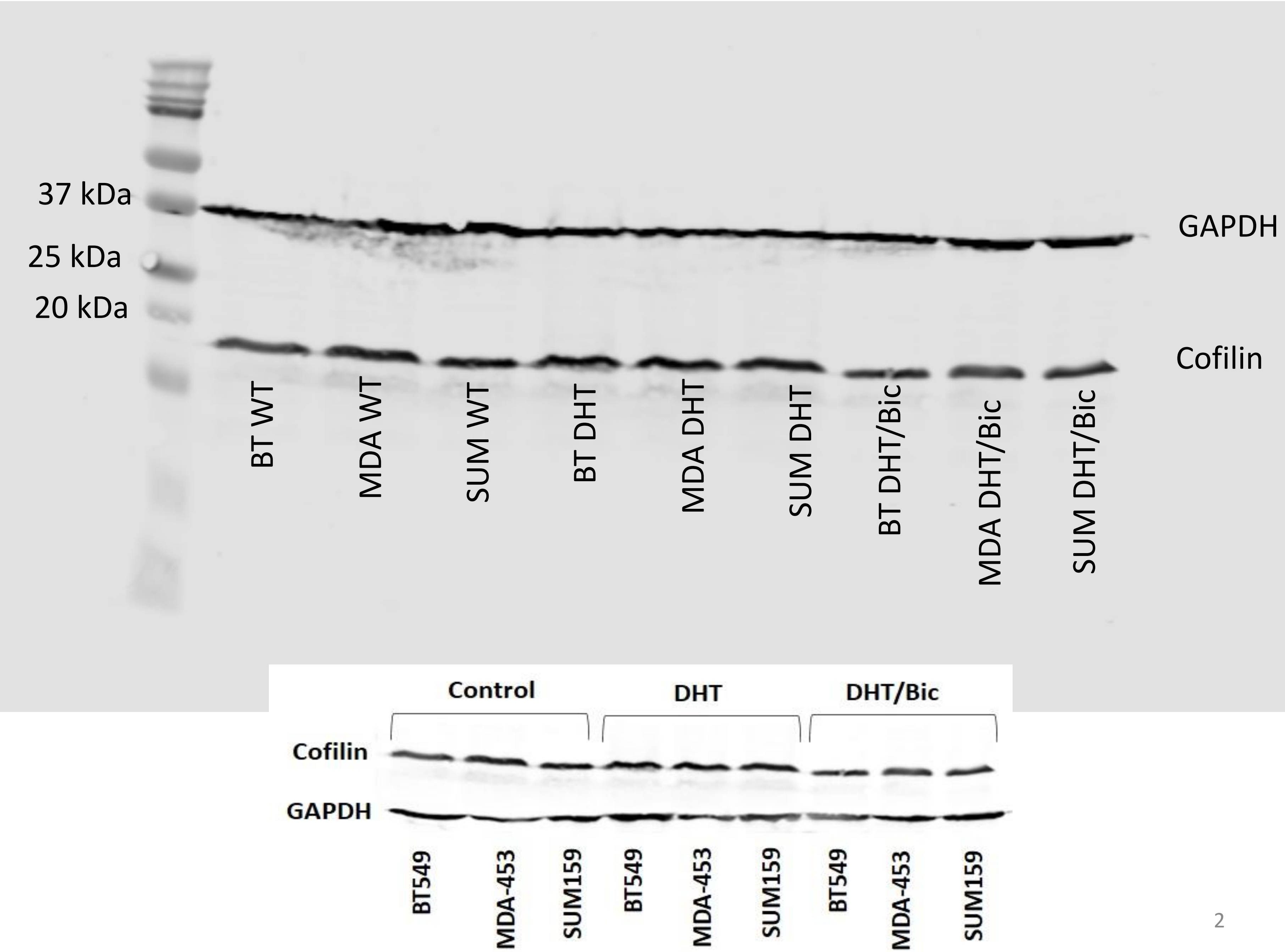
37 kDa
25 kDa
20 kDa
GAPDH
Cofilin
BT WT
MDA WT
SUM WT
BT DHT
MDA DHT
SUM DHT
BT DHT/Bic
MDA DHT/Bic
SUM DHT/Bic
Control
DHT
DHT/Bic
Cofilin
GAPDH
BT549
MDA-453
SUM159
BT549
MDA-453
SUM159
BT549
MDA-453
SUM159

Figure 2A

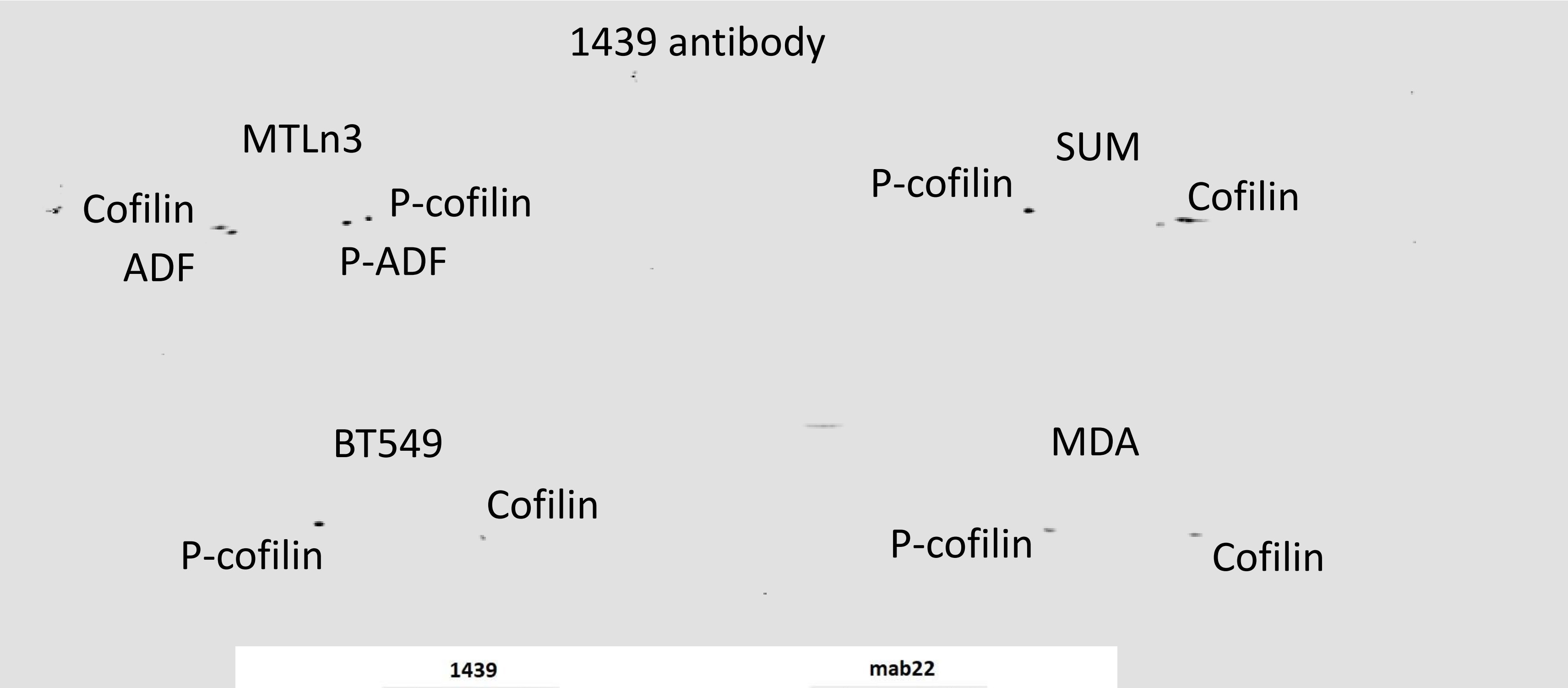

1439 antibody
MTLn3
Cofilin
P-cofilin
ADF
P-ADF
SUM
P-cofilin
Cofilin
BT549
Cofilin
P-cofilin
MDA
P-cofilin
Cofilin

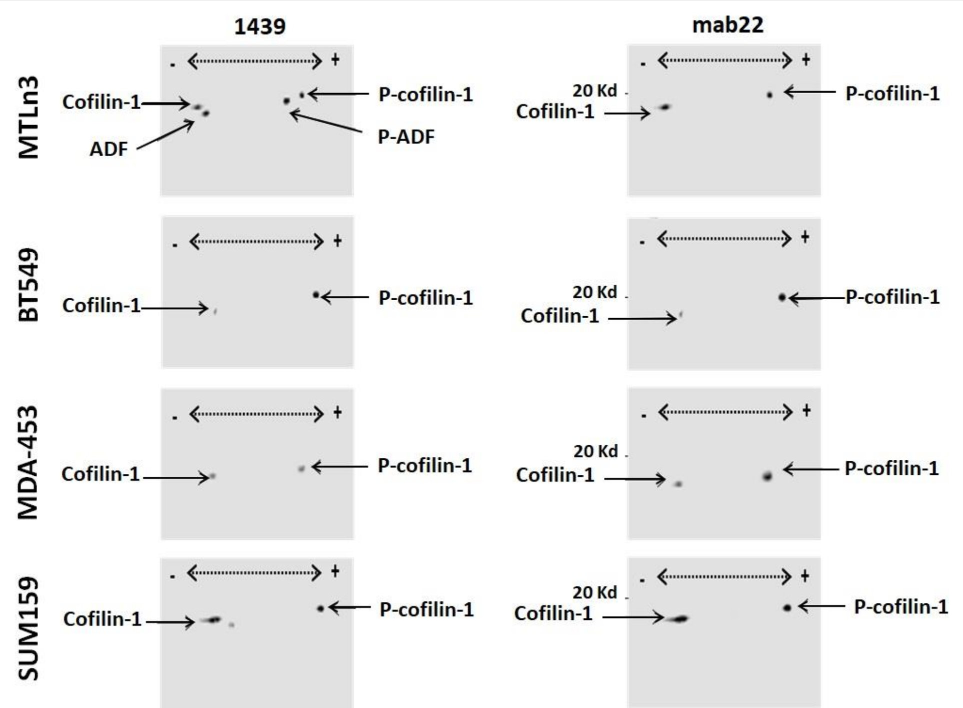

1439
mab22
MTLn3
Cofilin-1
P-cofilin-1
ADF
P-ADF
20 Kd
Cofilin-1
P-cofilin-1
BT549
Cofilin-1
P-cofilin-1
20 Kd
Cofilin-1
P-cofilin-1
MDA-453
Cofilin-1
P-cofilin-1
20 Kd
Cofilin-1
P-cofilin-1
SUM159
Cofilin-1
P-cofilin-1
20 Kd
Cofilin-1
P-cofilin-1

Figure 2A

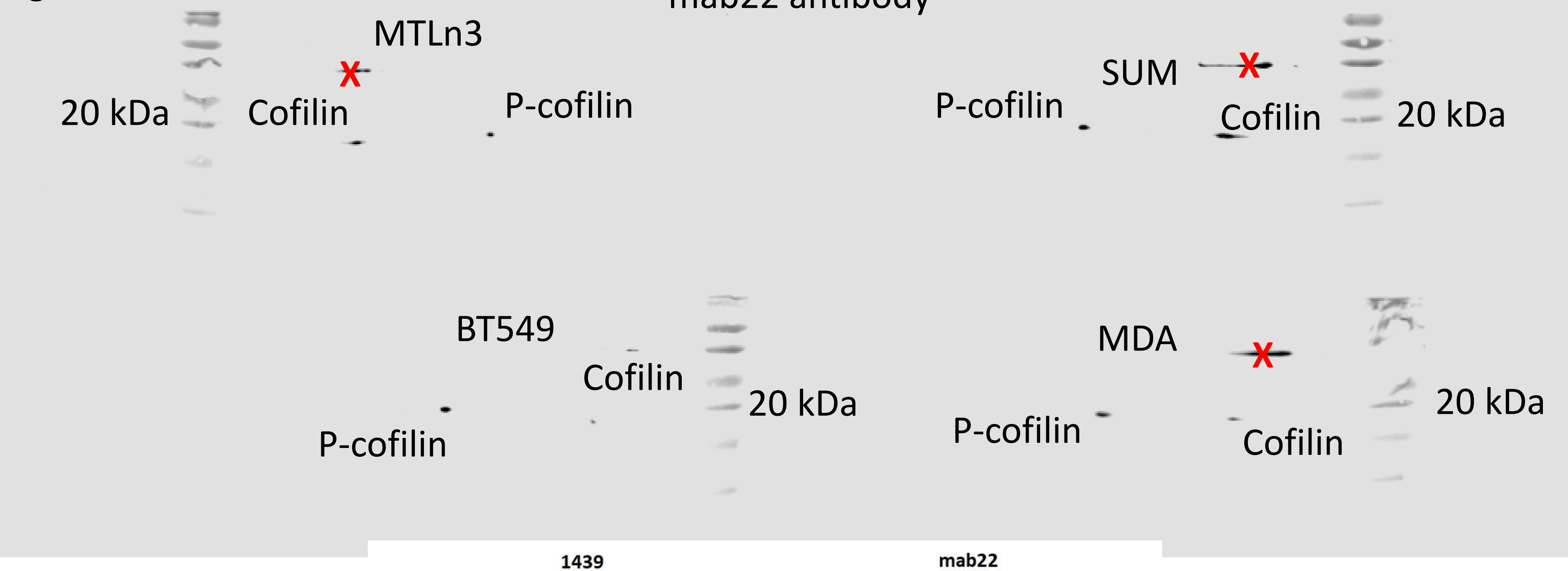
mab22 antibody
MTLn3
20 kDa
Cofilin
P-cofilin
SUM
P-cofilin
Cofilin
20 kDa
BT549
Cofilin
20 kDa
P-cofilin
MDA
P-cofilin
Cofilin
20 kDa

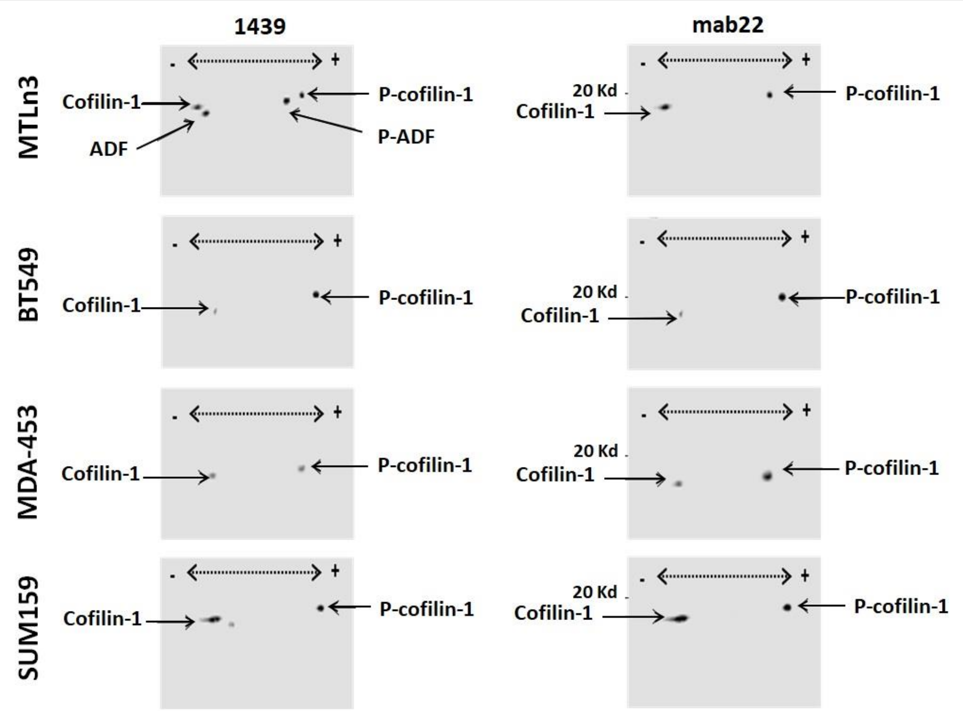
1439
mab22
MTLn3
Cofilin-1
ADF
P-cofilin-1
P-ADF
20 Kd
Cofilin-1
P-cofilin-1
BT549
Cofilin-1
P-cofilin-1
20 Kd
Cofilin-1
P-cofilin-1
MDA-453
Cofilin-1
P-cofilin-1
20 Kd
Cofilin-1
P-cofilin-1
SUM159
Cofilin-1
P-cofilin-1
20 Kd
Cofilin-1
P-cofilin-1

Figure 3A

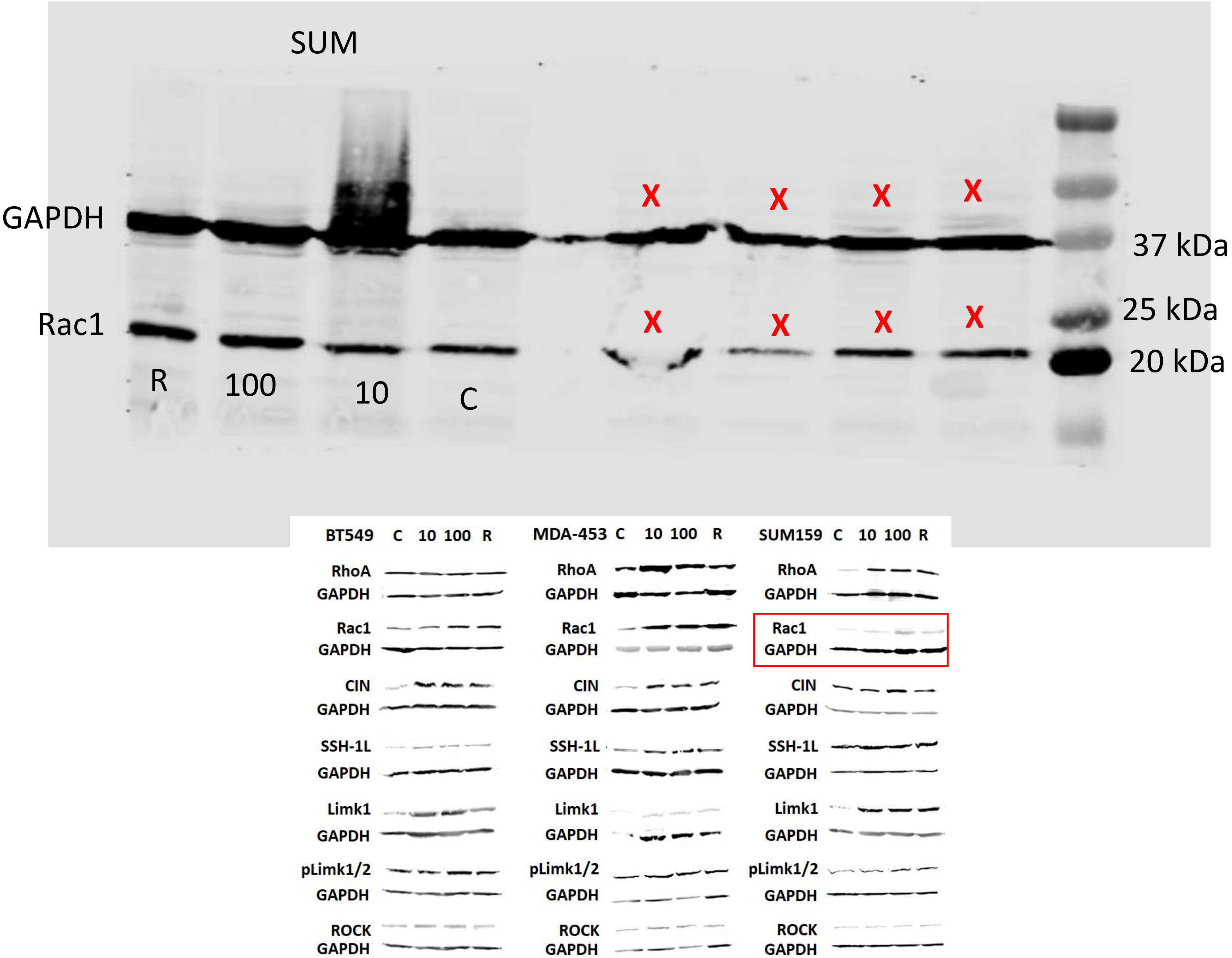
SUM
GAPDH
Rac1
R
100
10
C
X
X
X
X
X
X
X
X
37 kDa
25 kDa
20 kDa
BT549 C 10 100 R
MDA-453 C 10 100 R
SUM159 C 10 100 R
RhoA
GAPDH
Rac1
GAPDH
CIN
GAPDH
SSH-1L
GAPDH
Limk1
GAPDH
pLimk1/2
GAPDH
ROCK
GAPDH

Figure 3A

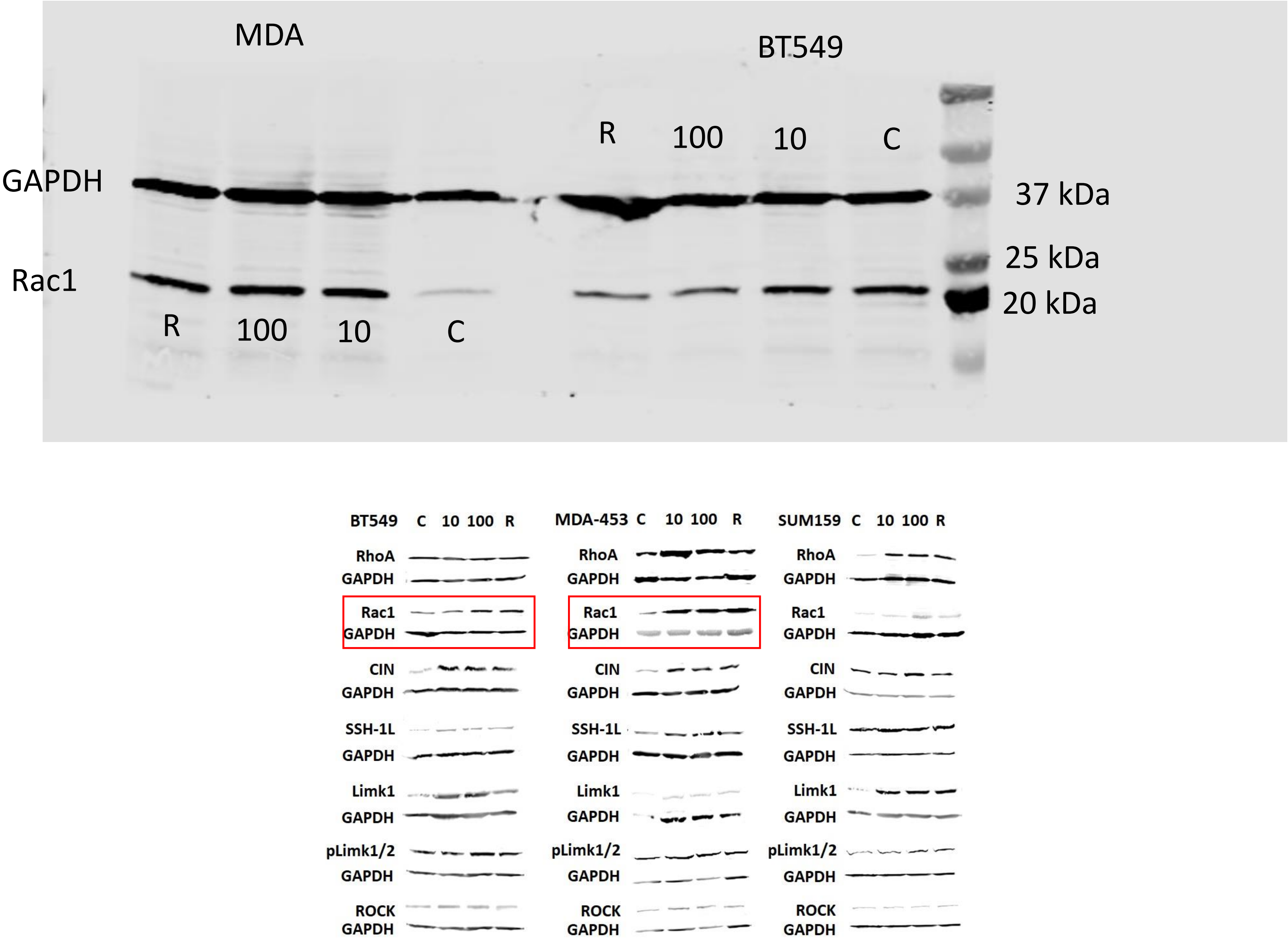

MDA
BT549
R
100
10
C
GAPDH
37 kDa
25 kDa
Rac1
20 kDa
R
100
10
C
BT549 C 10 100 R
MDA-453 C 10 100 R
SUM159 C 10 100 R
RhoA
GAPDH
Rac1
GAPDH
CIN
GAPDH
SSH-1L
GAPDH
Limk1
GAPDH
pLimk1/2
GAPDH
ROCK
GAPDH

Figure 3A

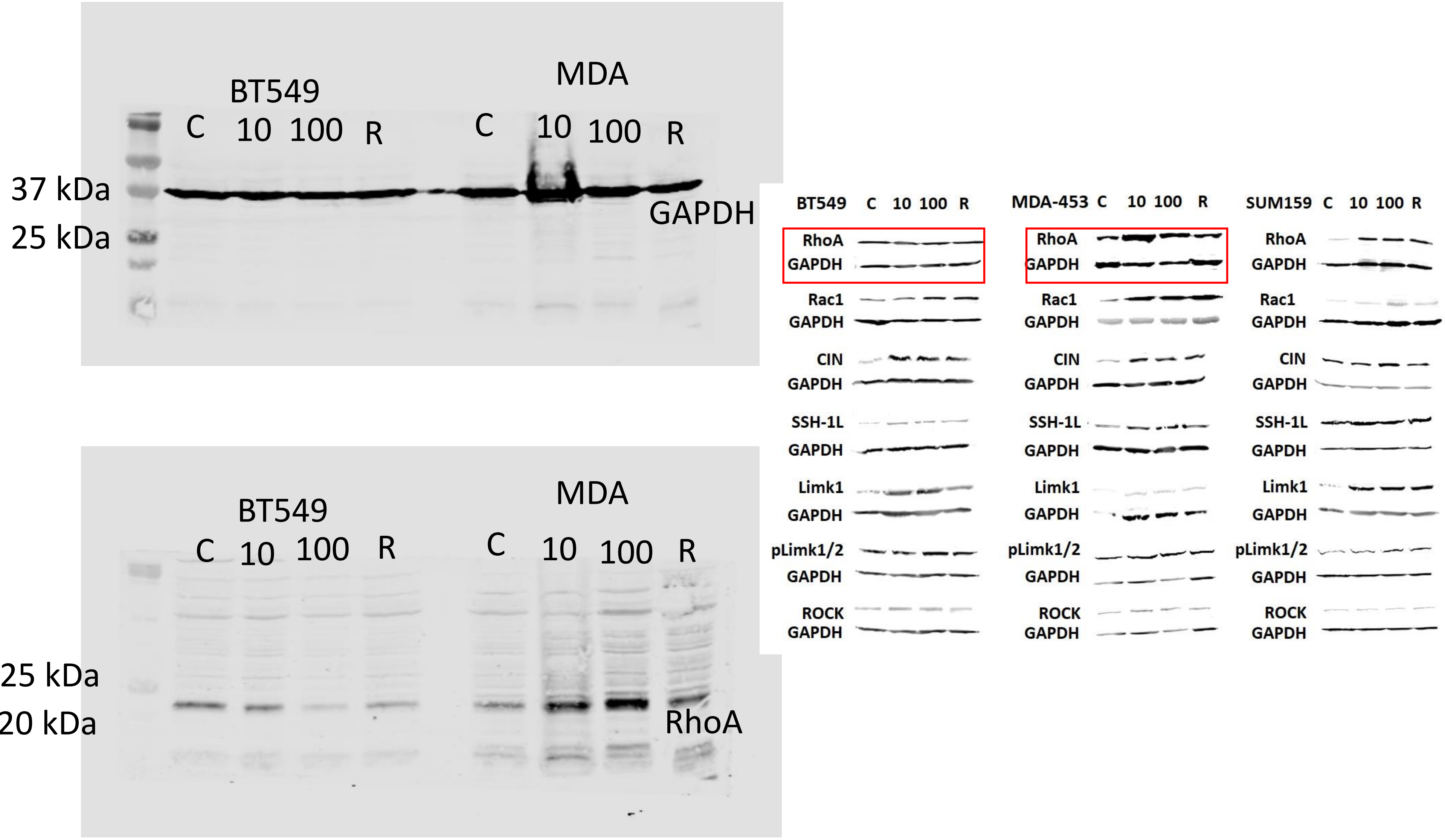

BT549
C 10 100 R
MDA
C 10 100 R
37 kDa
25 kDa
GAPDH
BT549
C 10 100 R
MDA
C 10 100 R
25 kDa
20 kDa
RhoA
BT549 C 10 100 R
MDA-453 C 10 100 R
SUM159 C 10 100 R
RhoA
GAPDH
Rac1
GAPDH
CIN
GAPDH
SSH-1L
GAPDH
Limk1
GAPDH
pLimk1/2
GAPDH
ROCK
GAPDH

Figure 3A

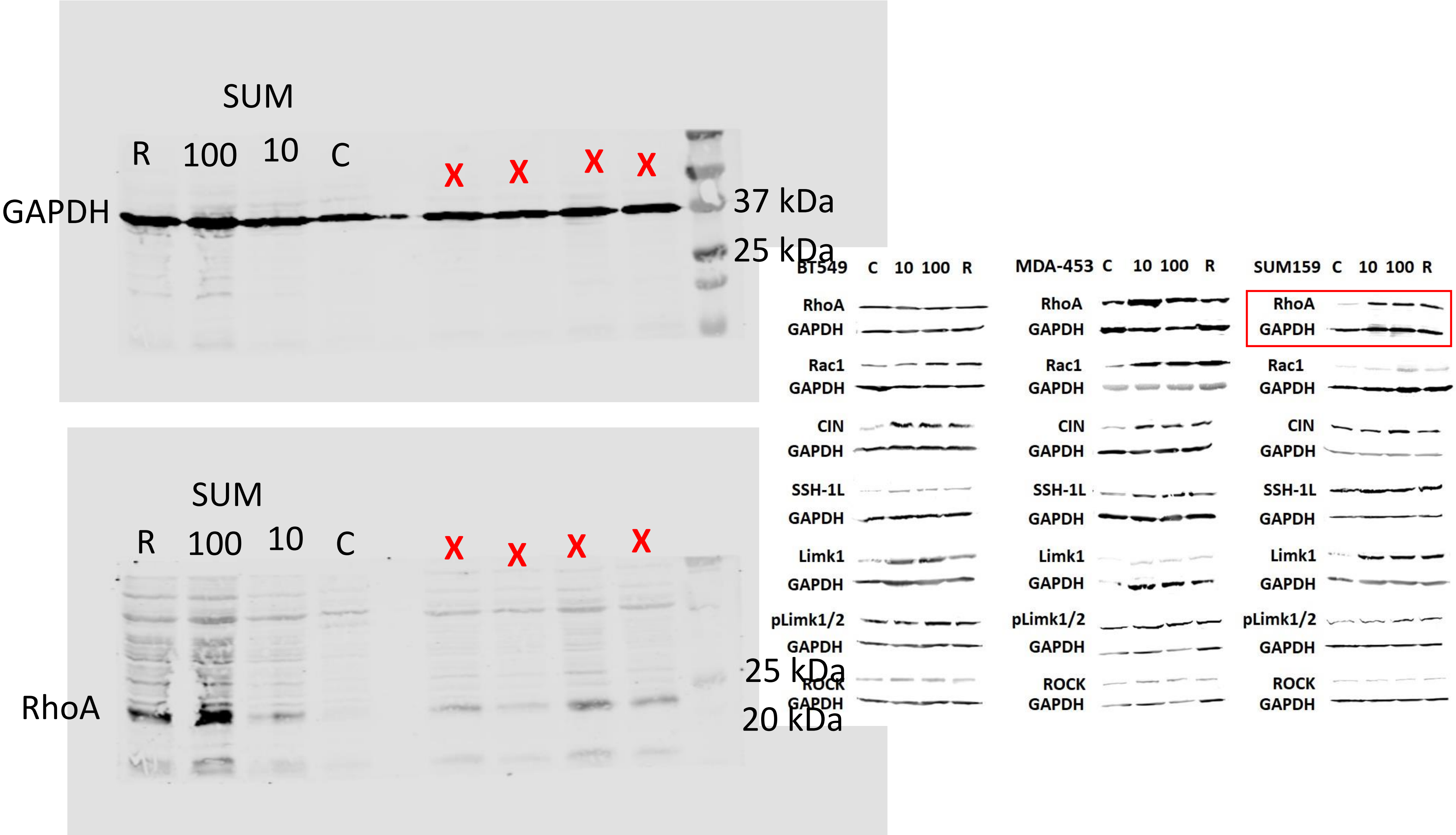
SUM
R 100 10 C
X X X X
GAPDH
37 kDa
25 kDa
SUM
R 100 10 C
X X X X
RhoA
25 kDa
20 kDa
BT549 C 10 100 R
MDA-453 C 10 100 R
SUM159 C 10 100 R
RhoA
GAPDH
Rac1
GAPDH
CIN
GAPDH
SSH-1L
GAPDH
Limk1
GAPDH
pLimk1/2
GAPDH
ROCK
GAPDH

Figure 3A

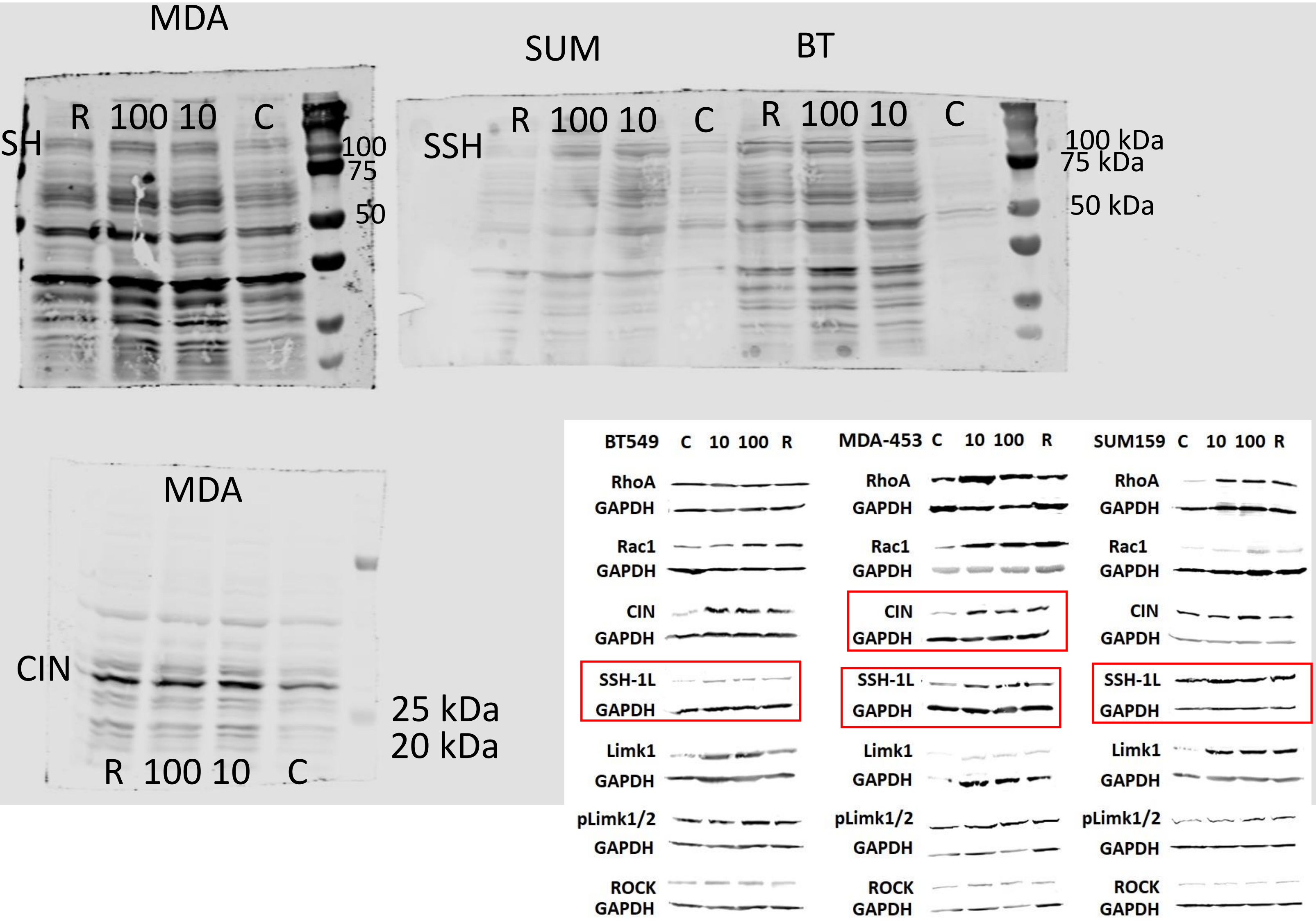

MDA
SUM
BT
SH
R 100 10 C
100
75
50
SSH
R 100 10 C
R 100 10 C
100 kDa
75 kDa
50 kDa
MDA
CIN
25 kDa
20 kDa
R 100 10 C
BT549 C 10 100 R
MDA-453 C 10 100 R
SUM159 C 10 100 R
RhoA
GAPDH
Rac1
GAPDH
CIN
GAPDH
SSH-1L
GAPDH
Limk1
GAPDH
pLimk1/2
GAPDH
ROCK
GAPDH

Figure 3A
GAPDH blots for blots on
slide 7

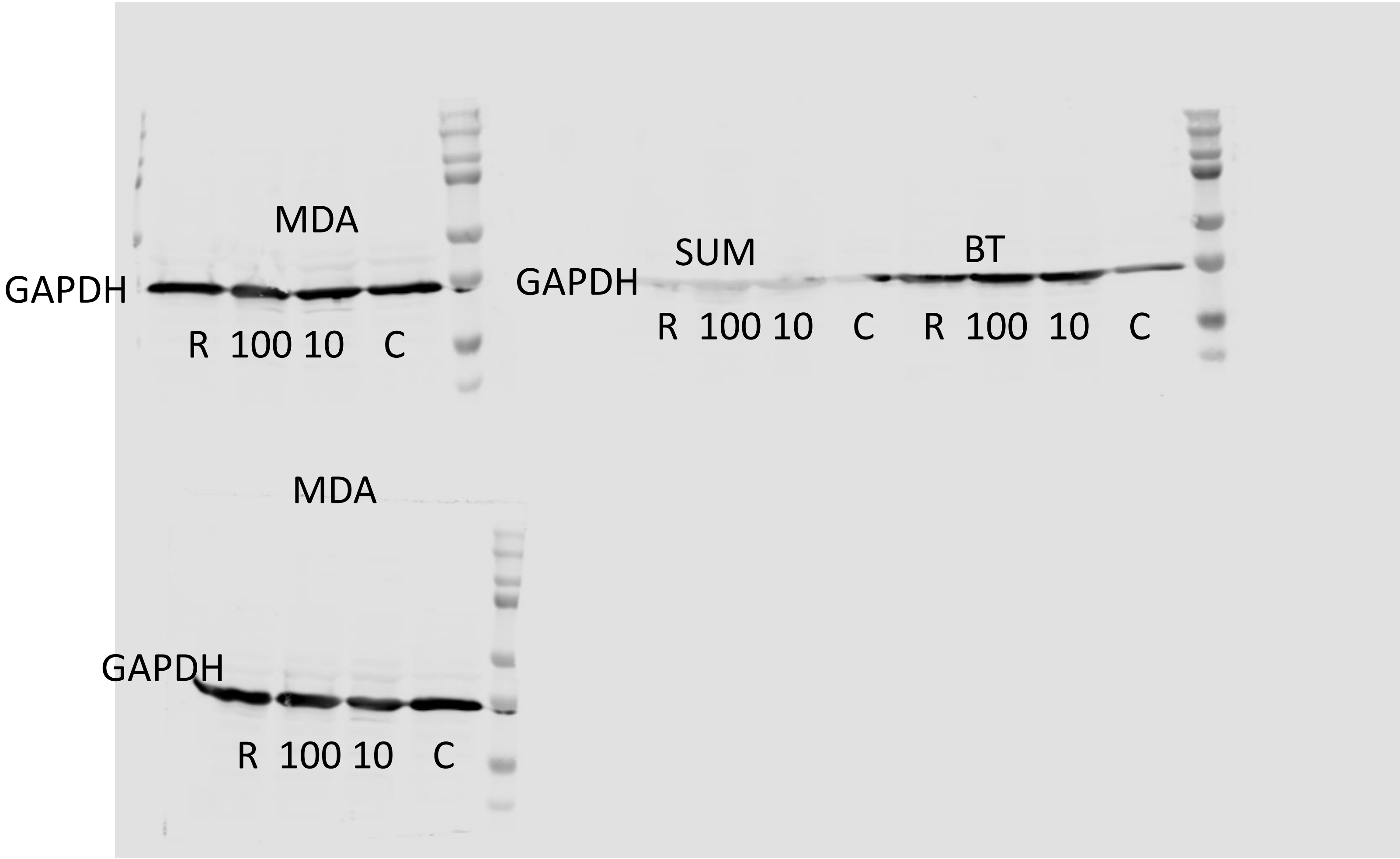

Figure 3A

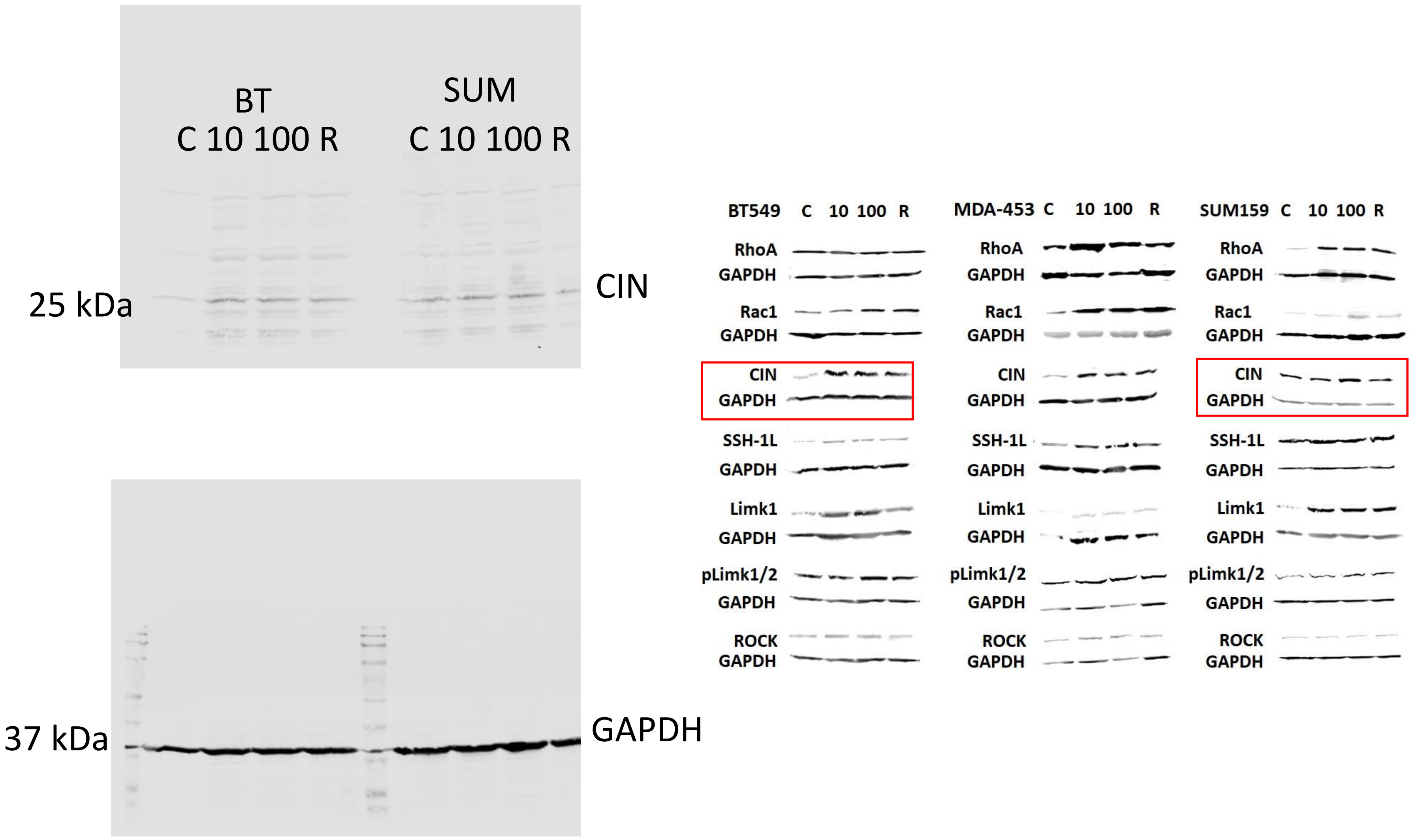
BT
C 10 100 R
SUM
C 10 100 R
25 kDa
CIN
37 kDa
GAPDH
BT549 C 10 100 R
MDA-453 C 10 100 R
SUM159 C 10 100 R
RhoA
GAPDH
Rac1
GAPDH
CIN
GAPDH
SSH-1L
GAPDH
Limk1
GAPDH
pLimk1/2
GAPDH
ROCK
GAPDH

Figure 3A

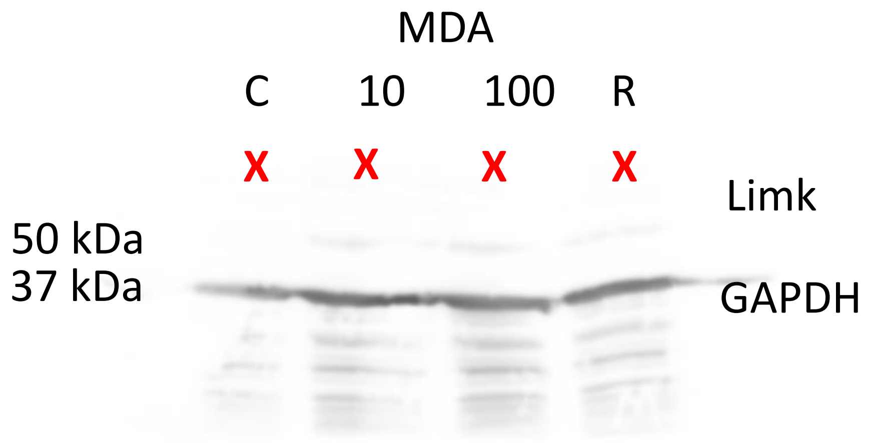
MDA
C
10
100
R
X
X
X
X
Limk
50 kDa
37 kDa
GAPDH

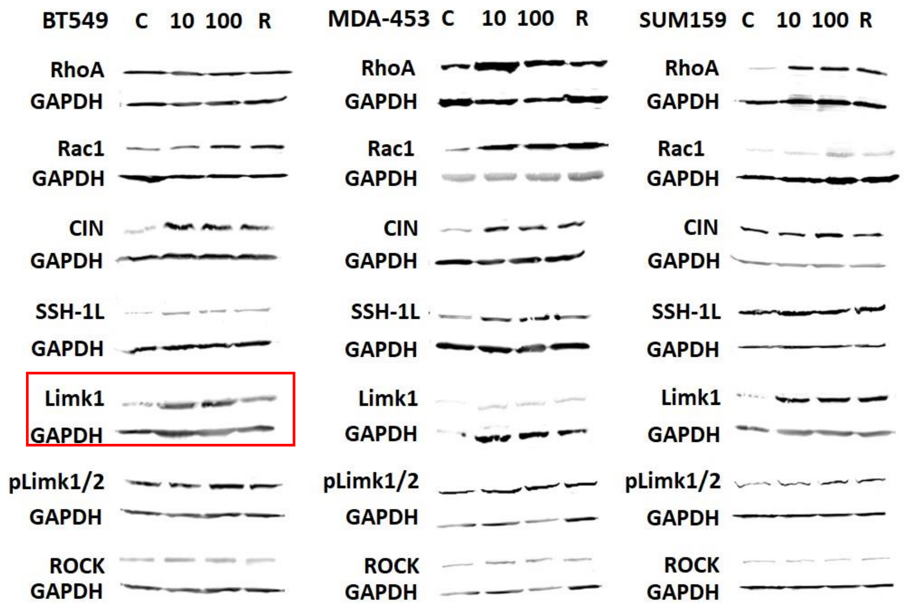
BT549 C 10 100 R
MDA-453 C 10 100 R
SUM159 C 10 100 R
RhoA
GAPDH
Rac1
GAPDH
CIN
GAPDH
SSH-1L
GAPDH
Limk1
GAPDH
pLimk1/2
GAPDH
ROCK
GAPDH

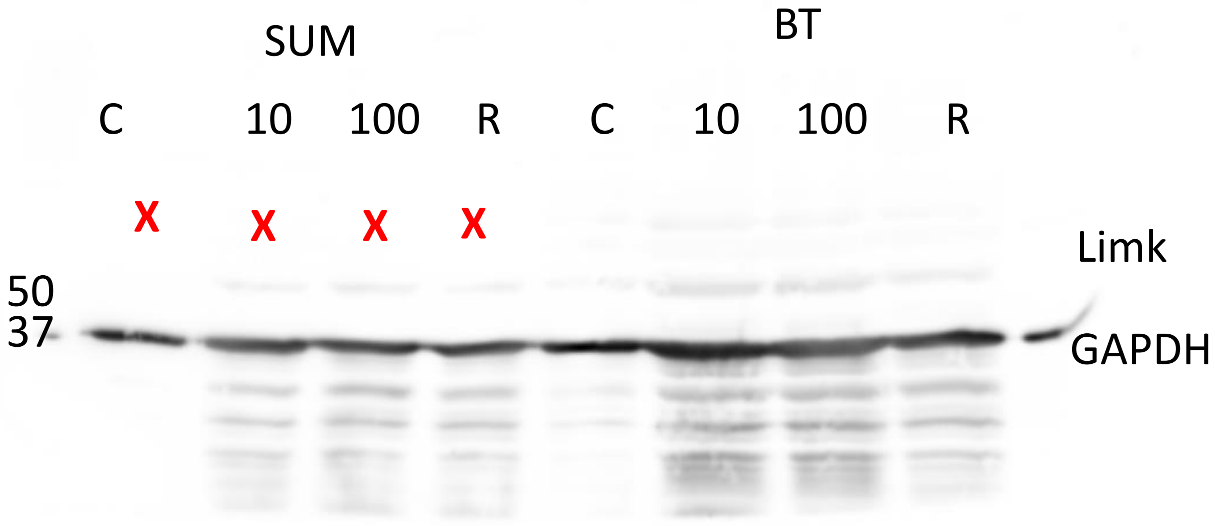
SUM
BT
C
10
100
R
C
10
100
R
X
X
X
X
Limk
50
37
GAPDH

Rat Limk1

MDA SUM

C 10 100 R C 10 100 R

Limk1

50 kDa

37 kDa

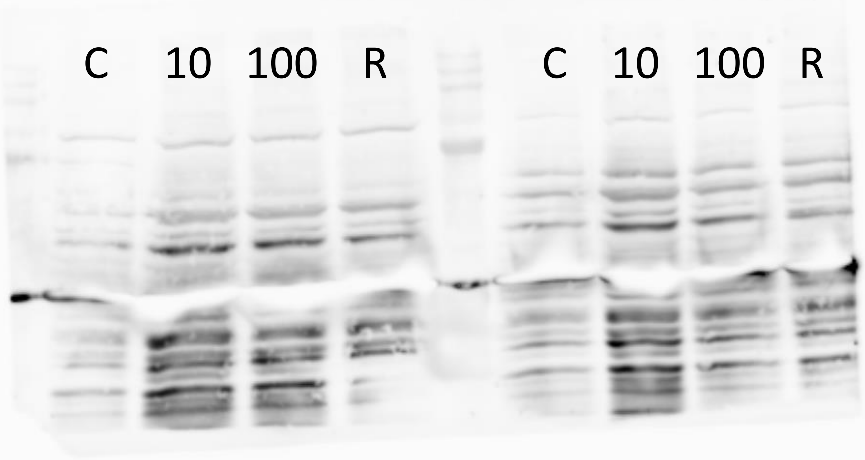

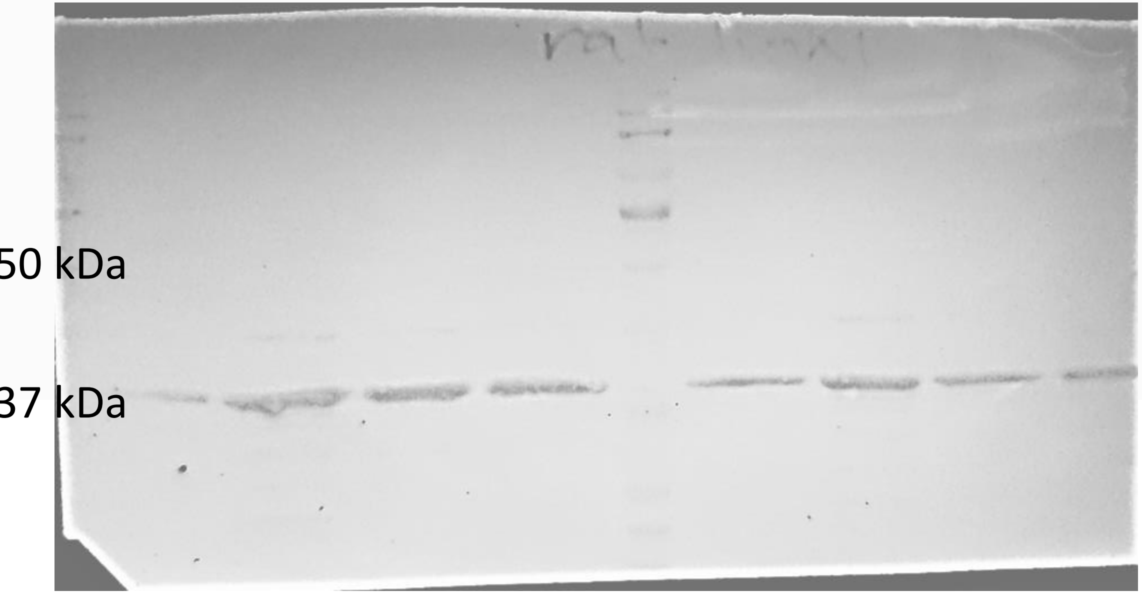

50 kDa

37 kDa

GAPDH

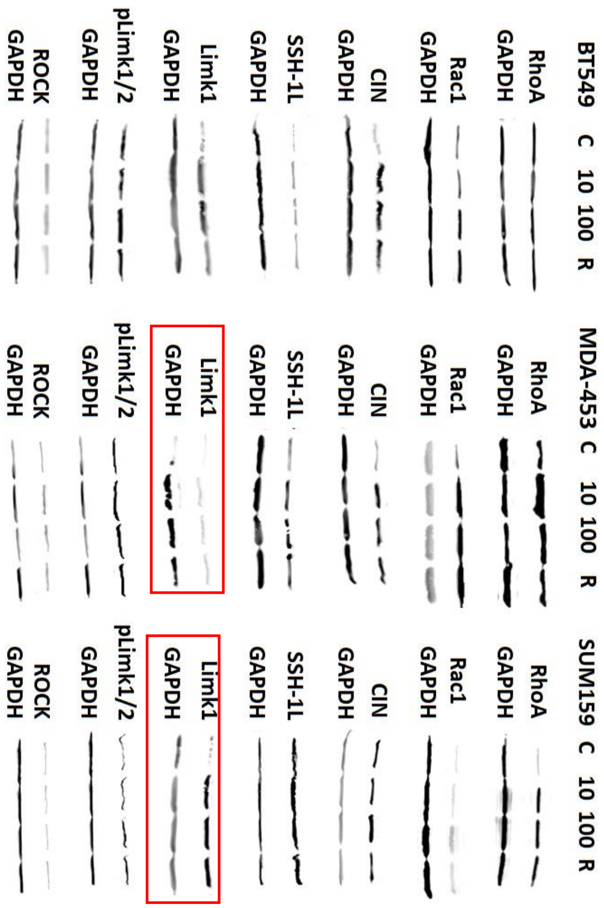

Figure 3A

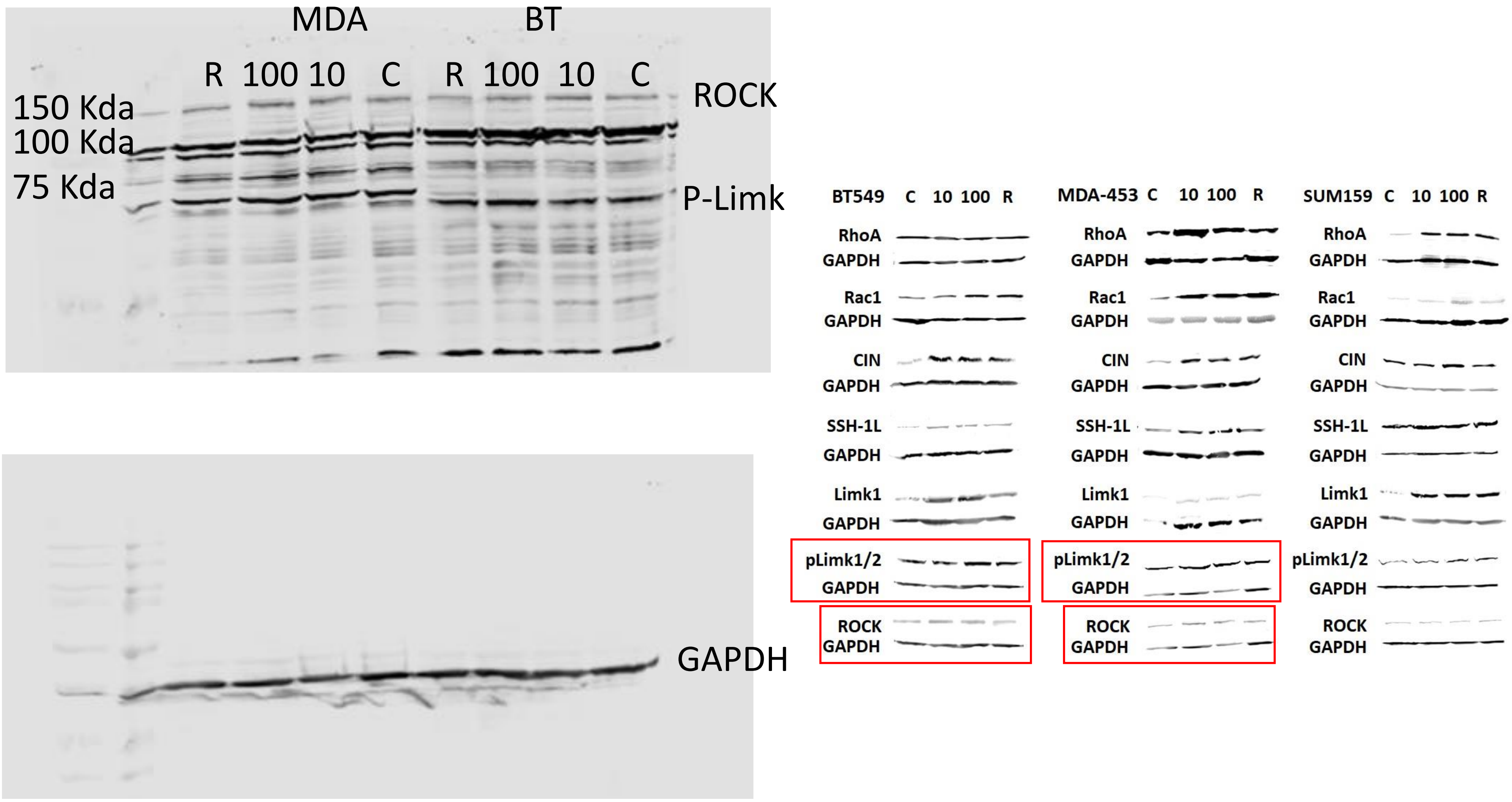
MDA
BT
R 100 10 C R 100 10 C
ROCK
150 Kda
100 Kda
75 Kda
P-Limk
GAPDH
BT549 C 10 100 R
MDA-453 C 10 100 R
SUM159 C 10 100 R
RhoA
GAPDH
Rac1
GAPDH
CIN
GAPDH
SSH-1L
GAPDH
Limk1
GAPDH
pLimk1/2
GAPDH
ROCK
GAPDH

Figure 3A

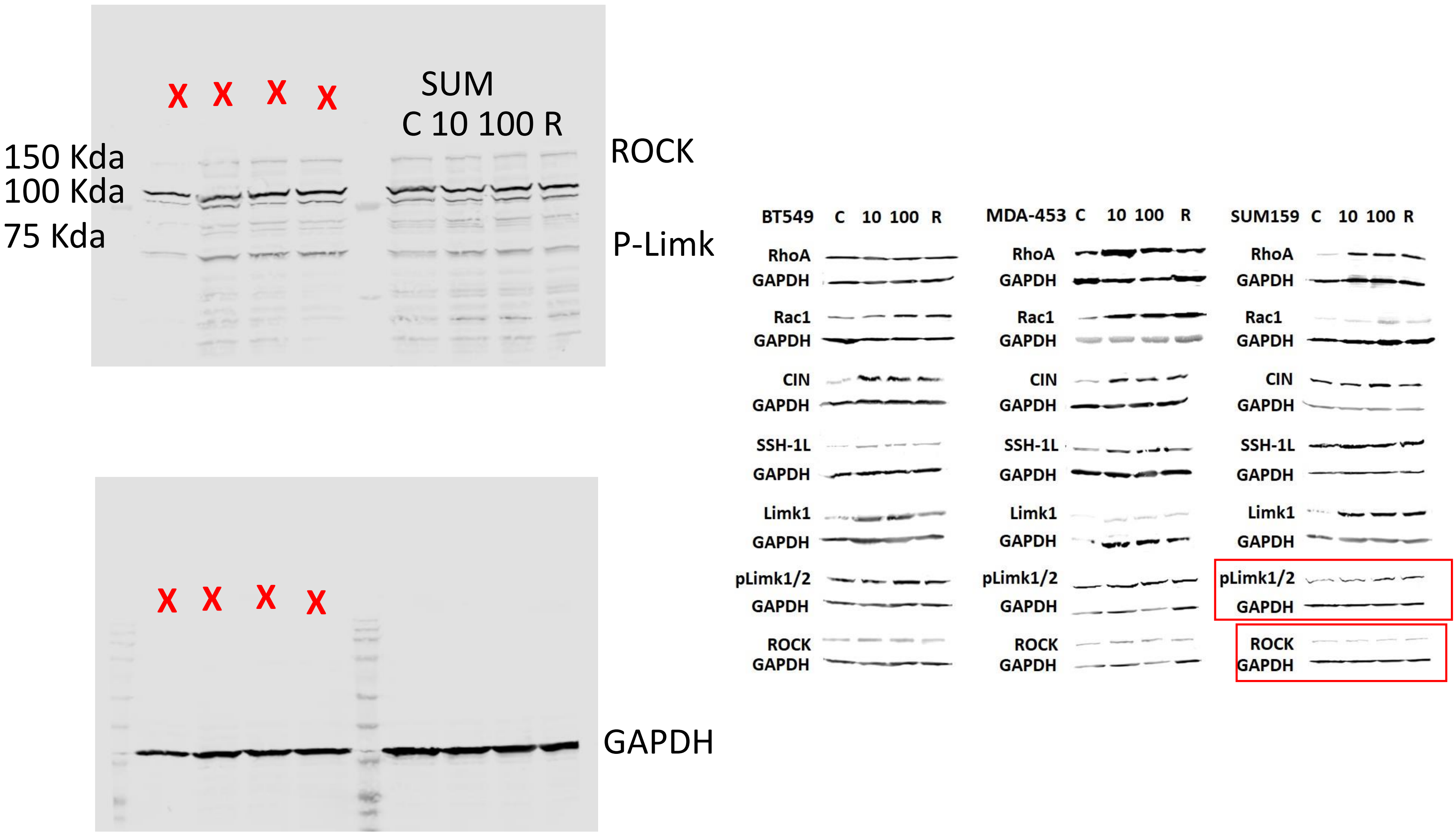
X X X X
SUM
C 10 100 R
150 Kda
100 Kda
75 Kda
ROCK
P-Limk
X X X X
GAPDH
BT549 C 10 100 R
MDA-453 C 10 100 R
SUM159 C 10 100 R
RhoA
GAPDH
Rac1
GAPDH
CIN
GAPDH
SSH-1L
GAPDH
Limk1
GAPDH
pLimk1/2
GAPDH
ROCK
GAPDH

Figure S1

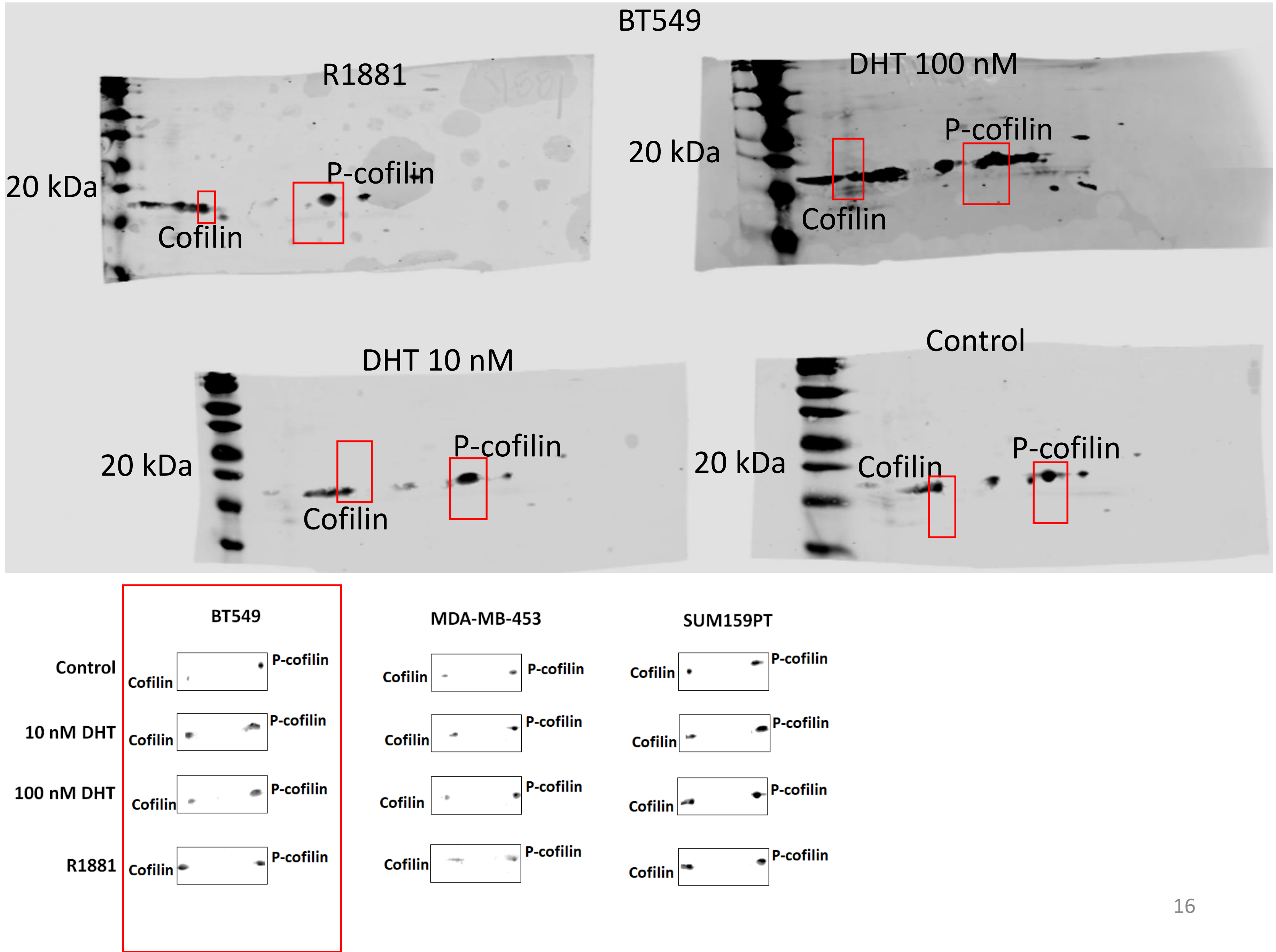
BT549
R1881
20 kDa
P-cofilin
Cofilin
DHT 100 nM
20 kDa
P-cofilin
Cofilin
DHT 10 nM
20 kDa
P-cofilin
Cofilin
Control
20 kDa
Cofilin
P-cofilin
BT549
MDA-MB-453
SUM159PT
Control
10 nM DHT
100 nM DHT
R1881
Cofilin
P-cofilin

Figure S1

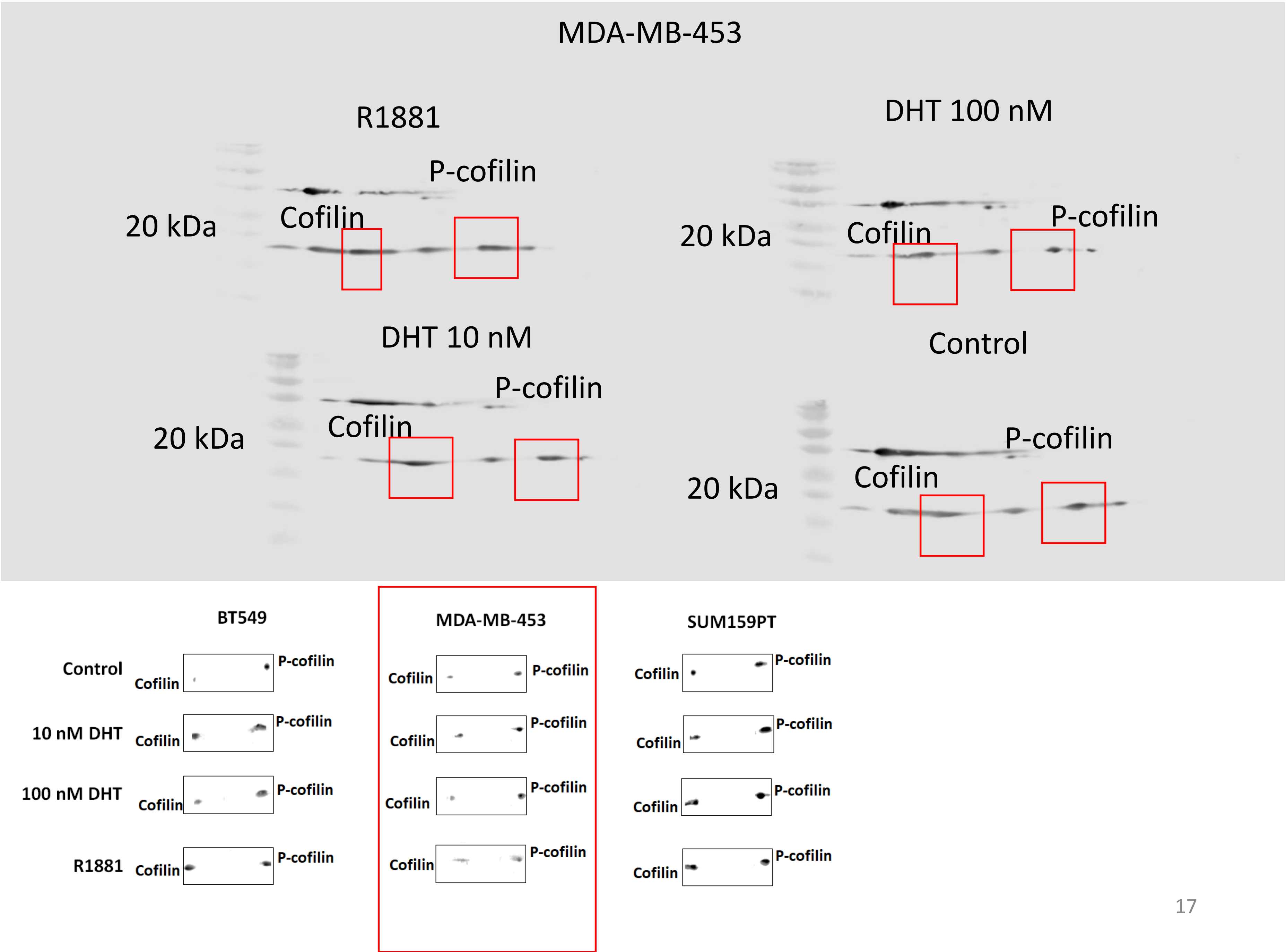
MDA-MB-453
R1881
P-cofilin
20 kDa
Cofilin
DHT 100 nM
20 kDa
Cofilin
P-cofilin
DHT 10 nM
P-cofilin
20 kDa
Cofilin
Control
P-cofilin
Cofilin
20 kDa
BT549
Control
Cofilin
P-cofilin
10 nM DHT
Cofilin
P-cofilin
100 nM DHT
Cofilin
P-cofilin
R1881
Cofilin
P-cofilin
MDA-MB-453
Cofilin
P-cofilin
Cofilin
P-cofilin
Cofilin
P-cofilin
Cofilin
P-cofilin
SUM159PT
Cofilin
P-cofilin
Cofilin
P-cofilin
Cofilin
P-cofilin
Cofilin
P-cofilin

Figure S1

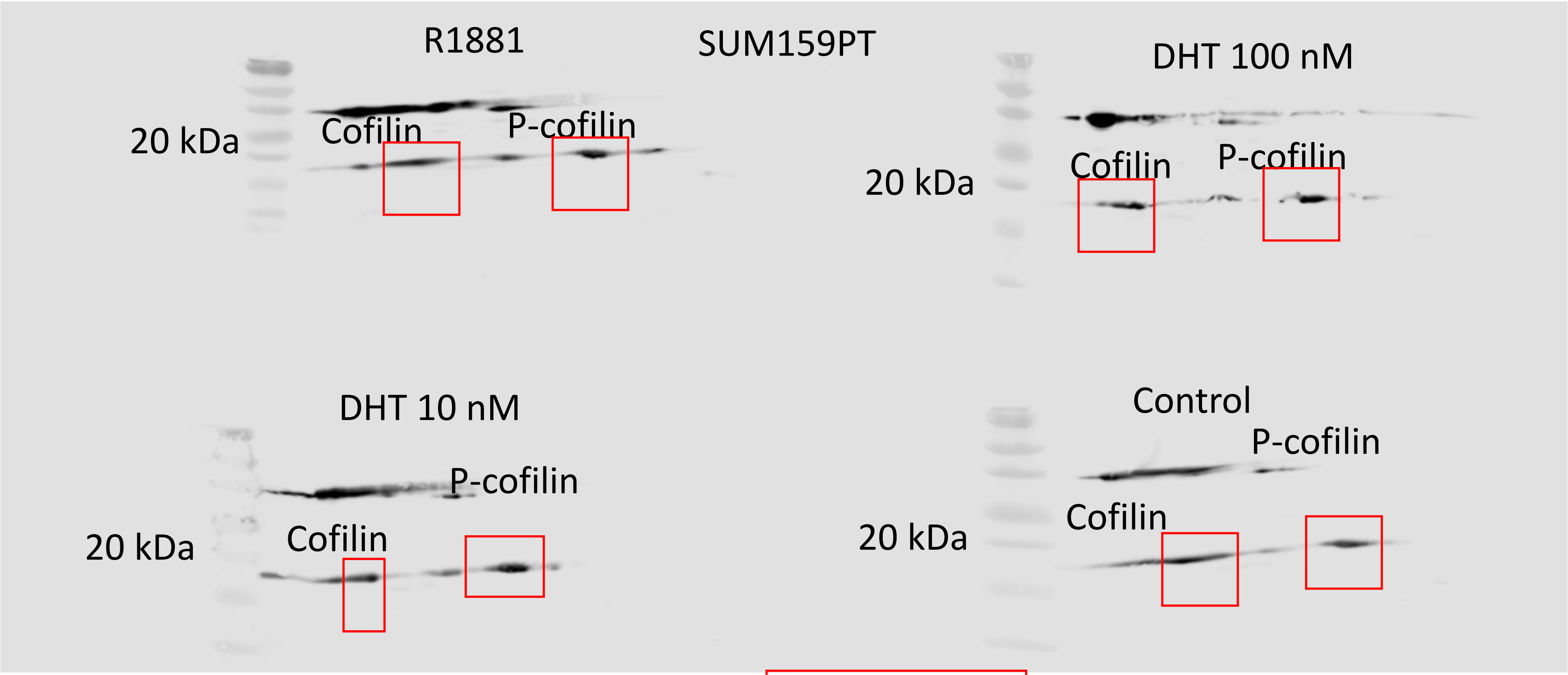

R1881
SUM159PT
20 kDa
Cofilin
P-cofilin
DHT 100 nM
20 kDa
Cofilin
P-cofilin
DHT 10 nM
P-cofilin
20 kDa
Cofilin
Control
P-cofilin
Cofilin
20 kDa

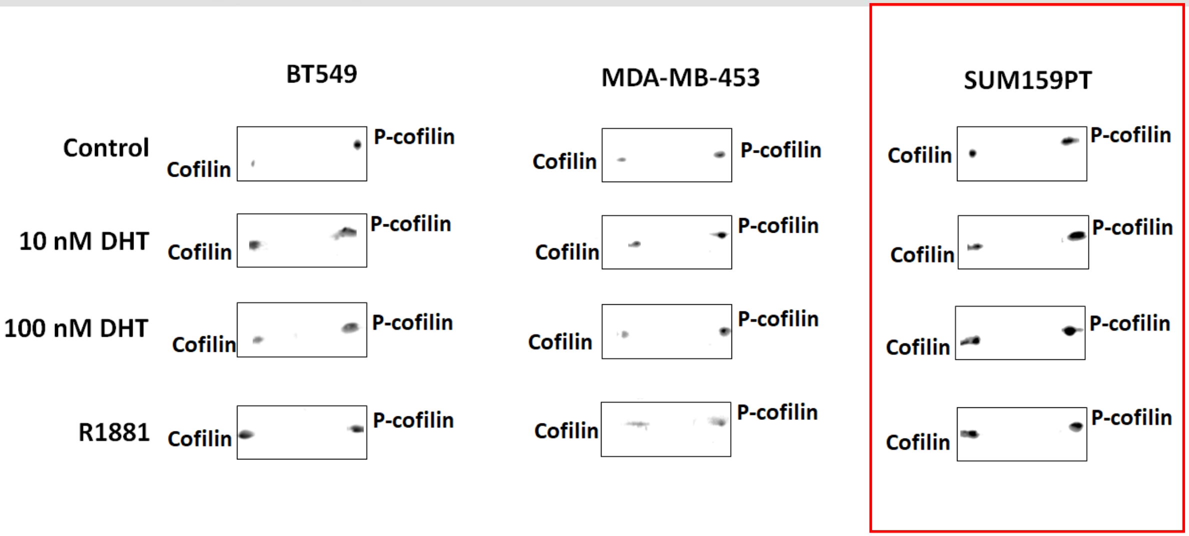

BT549
MDA-MB-453
SUM159PT
Control
10 nM DHT
100 nM DHT
R1881
Cofilin
P-cofilin

Figure S2A

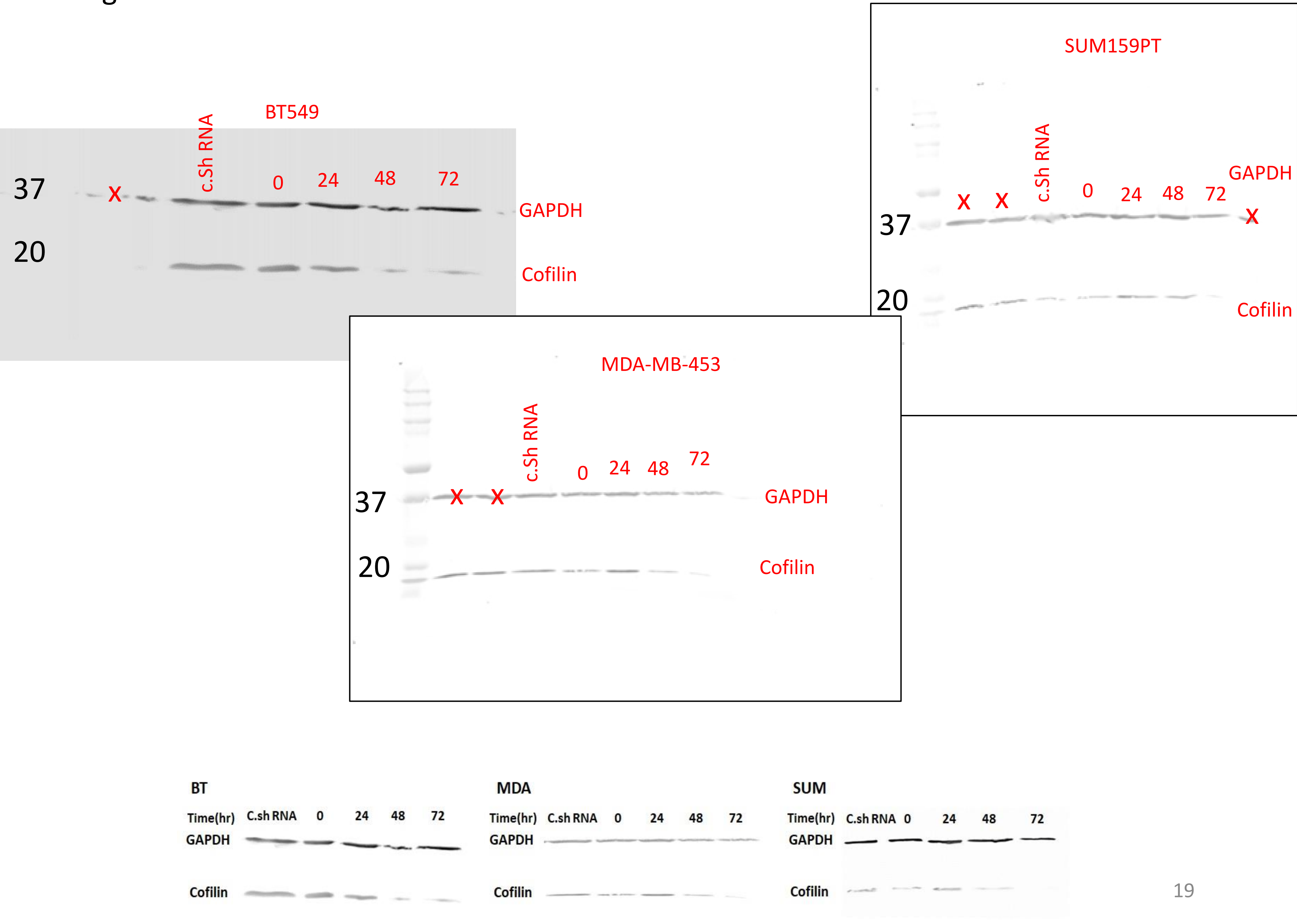
BT549
c.Sh RNA
0
24
48
72
37
20
X
GAPDH
Cofilin
SUM159PT
c.Sh RNA
0
24
48
72
37
20
X
X
X
GAPDH
Cofilin
MDA-MB-453
c.Sh RNA
0
24
48
72
37
20
X
X
GAPDH
Cofilin
BT
Time(hr) C.sh RNA 0 24 48 72
GAPDH
Cofilin
MDA
Time(hr) C.sh RNA 0 24 48 72
GAPDH
Cofilin
SUM
Time(hr) C.sh RNA 0 24 48 72
GAPDH
Cofilin

Figure S2B

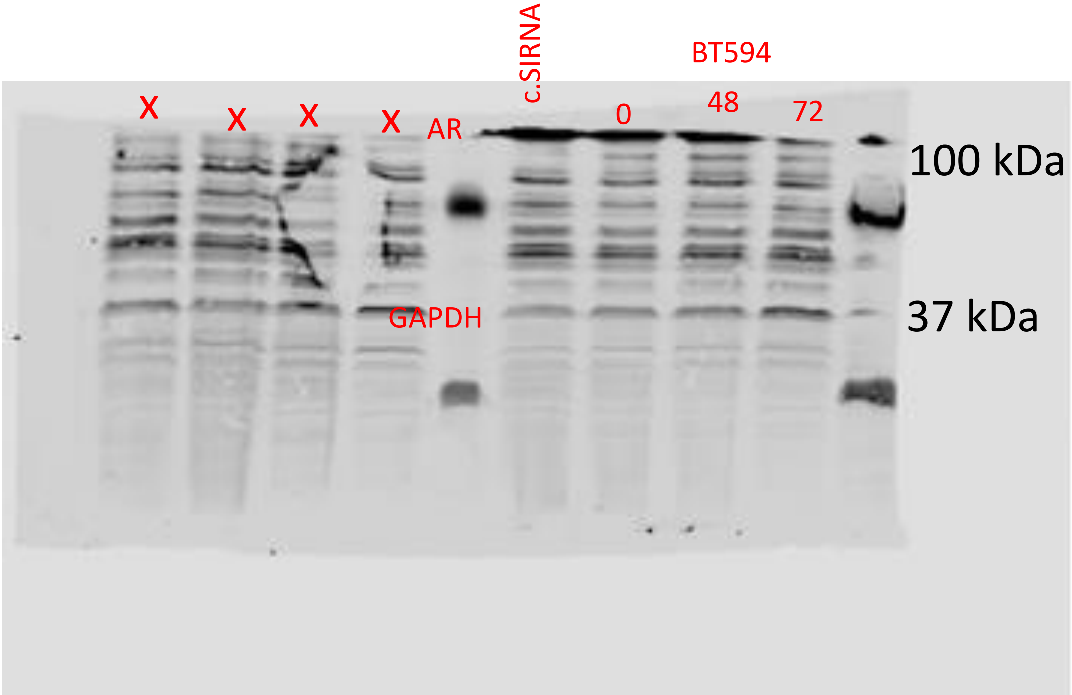
BT594
c.SIRNA
0
48
72
X
X
X
X
AR
GAPDH
100 kDa
37 kDa

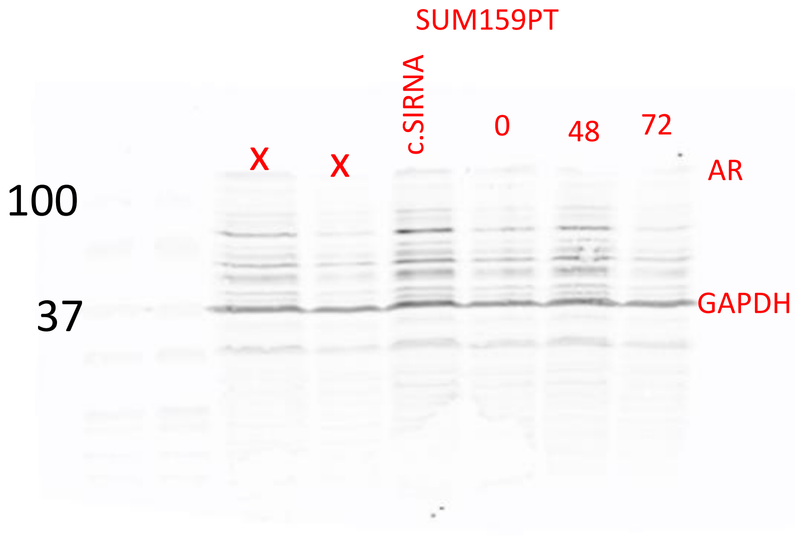
SUM159PT
c.SIRNA
0
48
72
X
X
AR
GAPDH
100
37

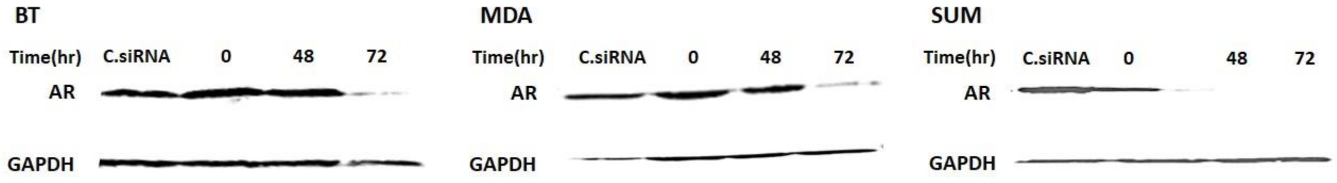
BT
Time(hr) C.siRNA 0 48 72
AR
GAPDH
MDA
Time(hr) C.siRNA 0 48 72
AR
GAPDH
SUM
Time(hr) C.siRNA 0 48 72
AR
GAPDH

Figure S2D

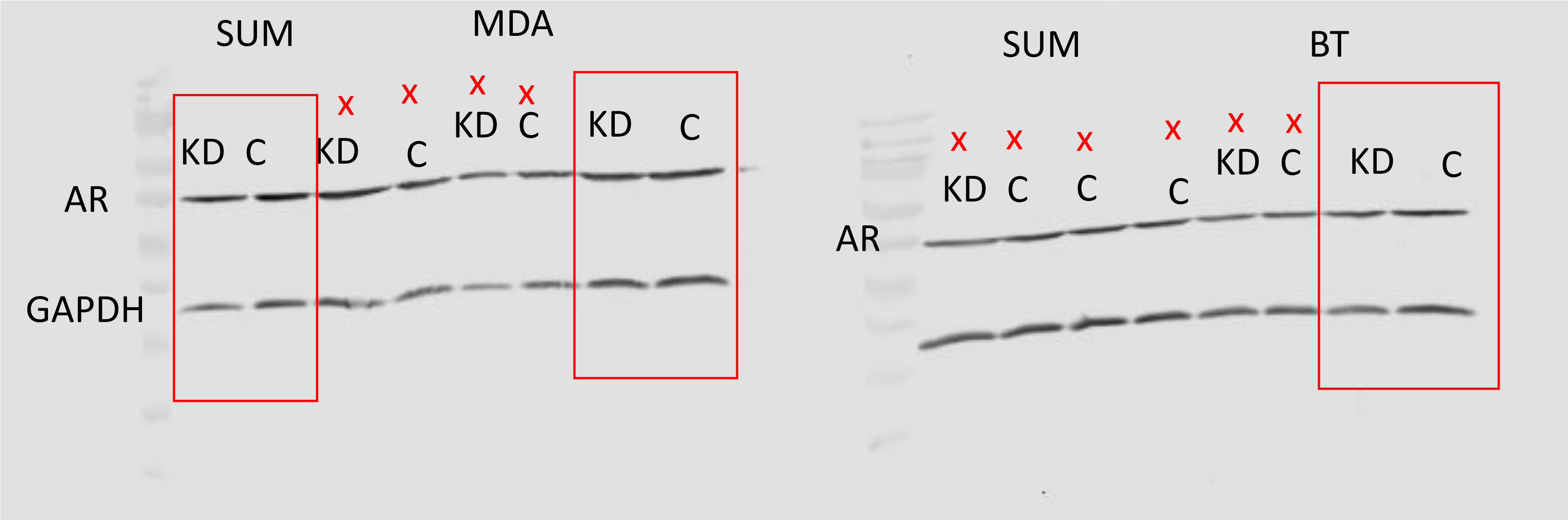

SUM
MDA
KD C
KD
C
KD C
KD C
AR
GAPDH
SUM
BT
KD C
C
C
KD C
KD C
AR

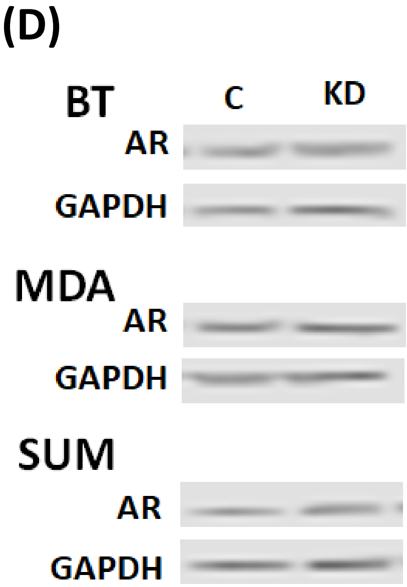

(D)
BT
C
KD
AR
GAPDH
MDA
AR
GAPDH
SUM
AR
GAPDH
