# Supplementary figures and images for: The role of activated androgen receptor in cofilin phospho-regulation depends on the molecular subtype of TNBC cell line and actin assembly dynamics
Source: PLoS One. 2022 Dec 30;17(12):e0279746. doi: 10.1371/journal.pone.0279746 (PMC9803305; doi:10.1371/journal.pone.0279746)

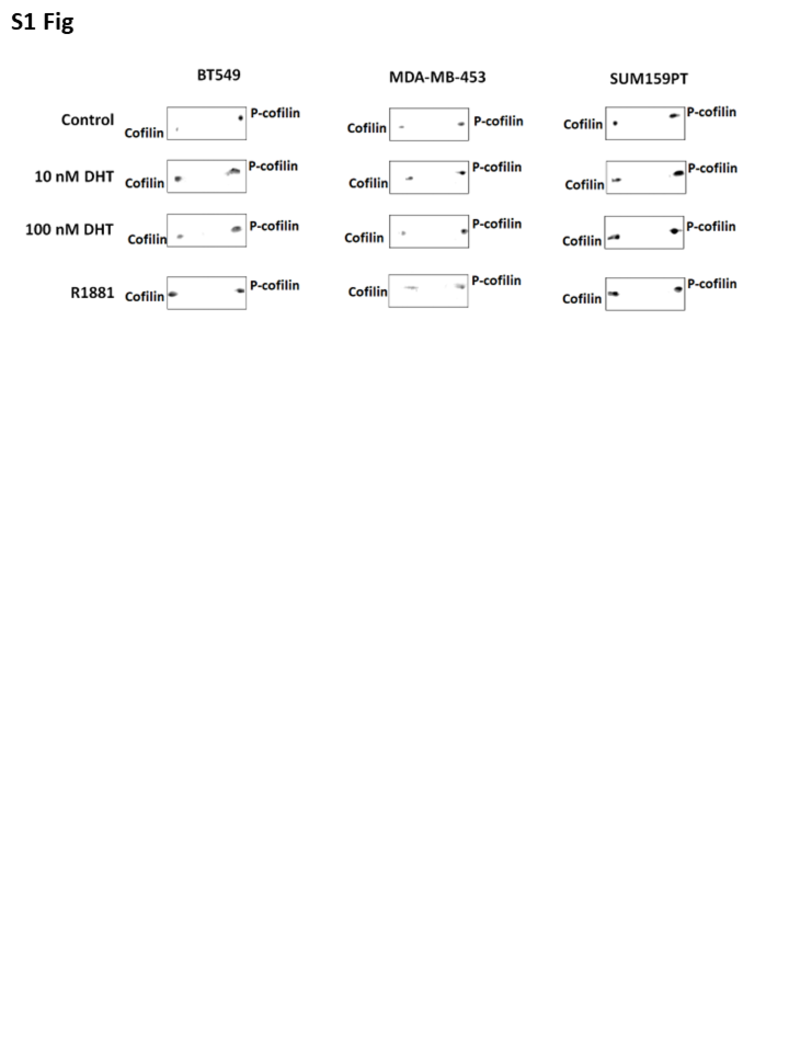

Supplement: S1 Fig — (TIF) [file pone.0279746.s003.tif]

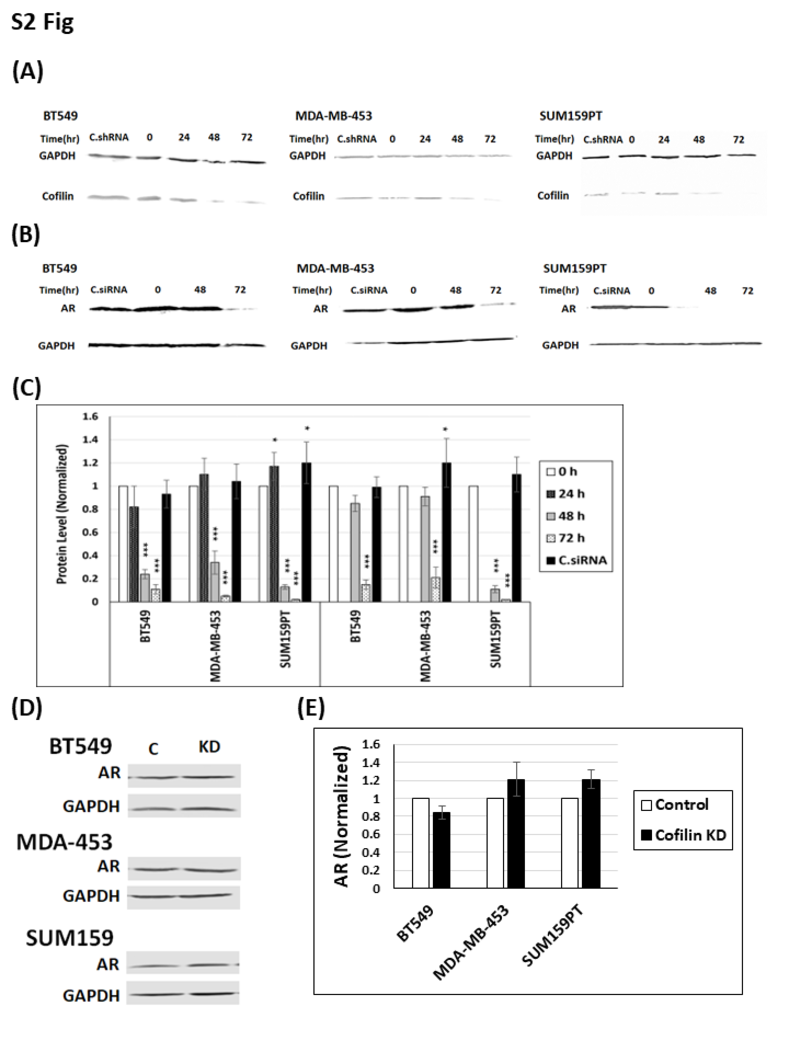

Supplement: S2 Fig — (A) Representative western blots of lysates from cells infected with adenovirus expressing control non-silencing shRNA (C.shRNA) and cells infected with adenovirus expressing cofilin shRNA. The experiment was repeated three times and the corresponding quantification is shown in (C). (B) Representative western blots of lysates from cells transfected with a control non-silencing siRNA (C.siRNA), and cells transfected with silencing siRNA against AR. The experiment was repeated three times and the corresponding quantification is shown in (C). (C) Quantification of cofilin and AR protein levels in shRNA/siRNA infected/transfected-TNBC cells relative to their levels in C.shRNA-infected or C.siRNA-transfected cells (set as 1.0 a.u.) obtained from densitometers of immunoblots. (D) Representative western blots of lysates from cells infected for 72 hr with adenovirus for expressing cofilin shRNA (cofilin KD; KD in the Fig). Blots were immunolabeled using the AR antibody and a monoclonal antibody to GAPDH as a loading control. The experiment was repeated three times and the corresponding quantification is shown in (E). (E) Quantification of AR levels in cofilin KD cells as compared to control uninfected cells (set as 1.0) obtained from densitometers of immunoblots. (TIF) [file pone.0279746.s004.tif]

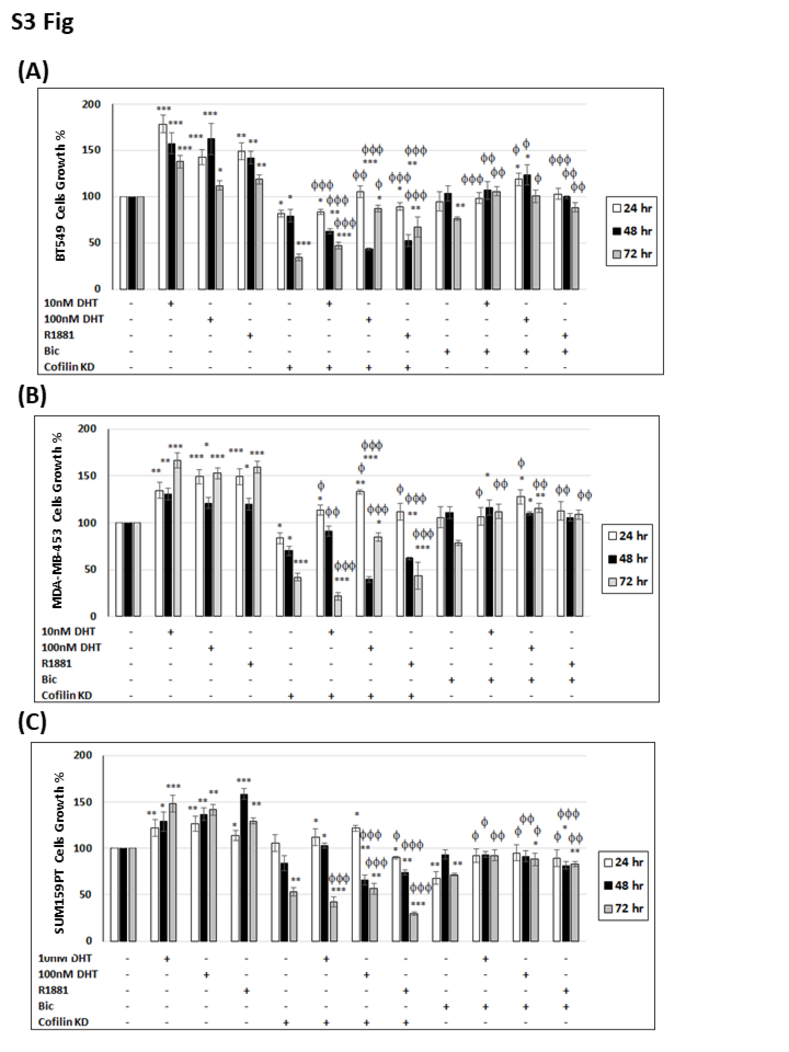

Supplement: S3 Fig — TNBC cells (A) BT549, (B) MDA-MB-453, and (C) SUM159PT were treated for 24, 48, and 72 hr and the growth percentage (Growth %) relative to the vehicle-treated control cells (set as 100%) was measured by MTT assay. Bars = mean ± SEM of three independent experiments performed in triplicates. * p <0.05, ** p <0.01, *** p <0.001 versus control, ϕ p < 0.05, ϕϕ p <0.01, ϕϕϕ p <0.001 versus androgen-treated cells. (TIF) [file pone.0279746.s005.tif]

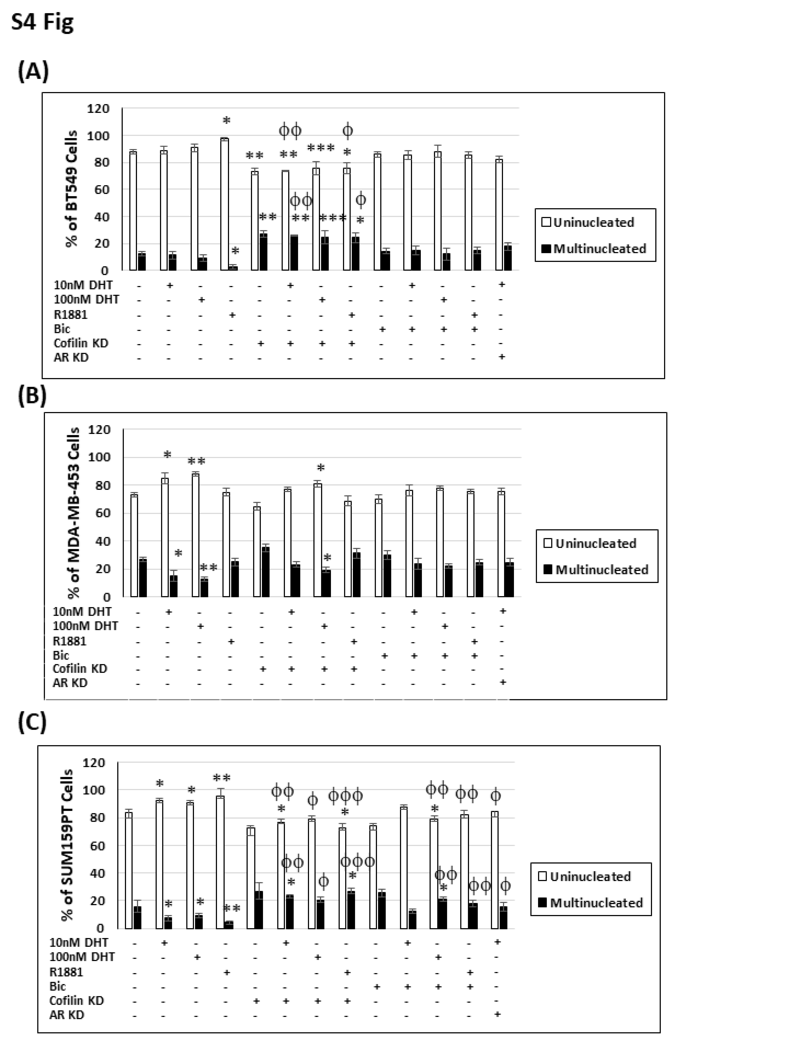

Supplement: S4 Fig — TNBC cells (A) BT549, (B) MDA-MB-453, and (C) SUM159PT were stained with DAPI and scored for the percentage of uninucleation and multinucleation. n ≥ 100 cells in each experiment, three independent experiments. * p<0.05, ** p< 0.01, *** p<0.001 versus control, ϕ p< 0.05, ϕϕ p<0.01, ϕϕϕ P< 0.001 versus androgen-treated cells. (TIF) [file pone.0279746.s006.tif]

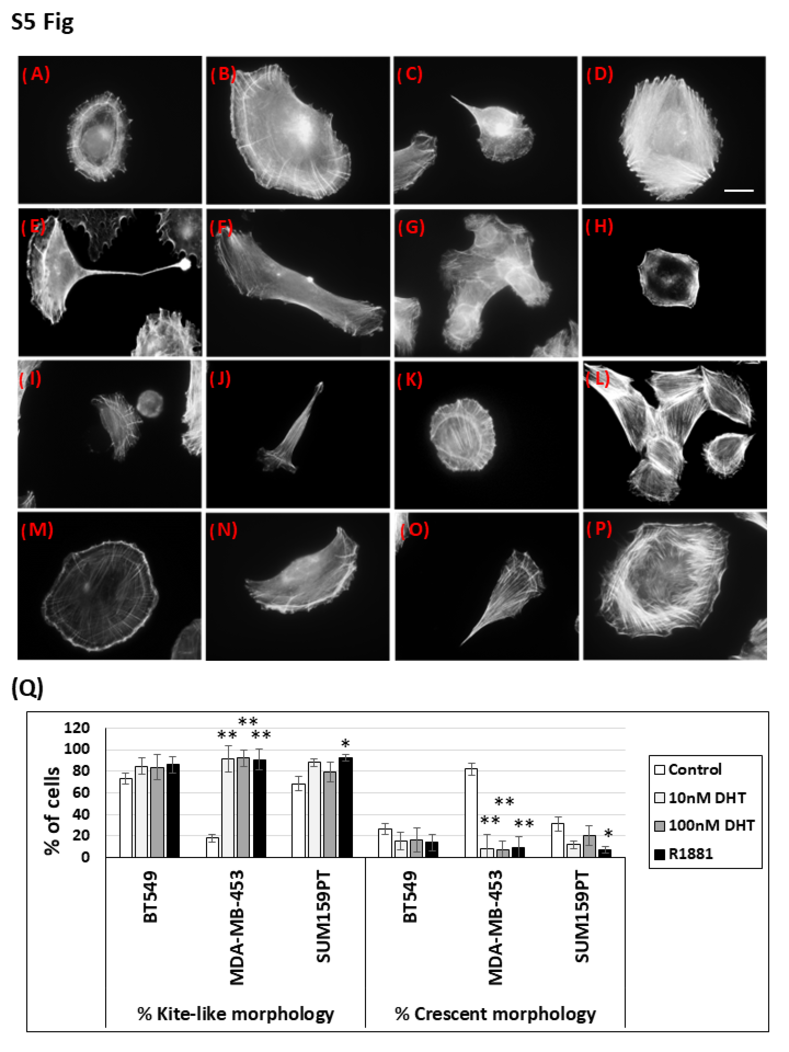

Supplement: S5 Fig — TNBC cells were stained with fluorescent-conjugated phalloidin. (A) Apolar BT549 cell displaying cuboidal epithelium morphology with cortical actin cytoskeleton. (B) Crescent polar BT549 cell. (C) Kite-like polar BT549 cell. (D) Cofilin shRNA-transfected BT549 cell showing thick stress fibers and cell rounding. (E) Androgen-treated BT549 cell showing a dramatic change in cell morphology with an elongated cytoplasmic extension that resembles a neurite. (F) Bipolar BT549 cell having two lamellipodia. (G) MDA-MB-453 cells grow as clusters (grape-like or stellate structures). (H) Individual MDA-MB-453 cell showing prominent cortical actin. (I) Crescent polar MDA-MB-453 cell. (J) Kite-like polar MDA-MB-453 cell. (K) Knocking down cofilin in MDA-MB-453 cells resulted in the formation of thick stress filaments and cell rounding (apolar fried-egg morphology). (L) SUM159PT cells grow mainly in groups. (M) Individual SUM159PT cell showing homogeneously distributed actin filament and prominent lamellipodium. (N) Crescent polar SUM159PT cell. (O) Kite-like polar SUM159PT cell. (P) SUM159PT cell expressing cofilin shRNA shows cell expansion and prominent actin stress fibers. Scale bar: 10 μm. (Q) Androgen-treated TNBC cells exhibit the kite-like morphology. Cells were stained with fluorescent-conjugated phalloidin and the percentage of polarized cells exhibiting the kite-like morphology, or the crescent morphology was scored. Data are expressed as mean ± SEM, n ≥ 100 cells in each experiment, three independent experiments. * p<0.05, ** p< 0.01 versus control. (TIF) [file pone.0279746.s007.tif]

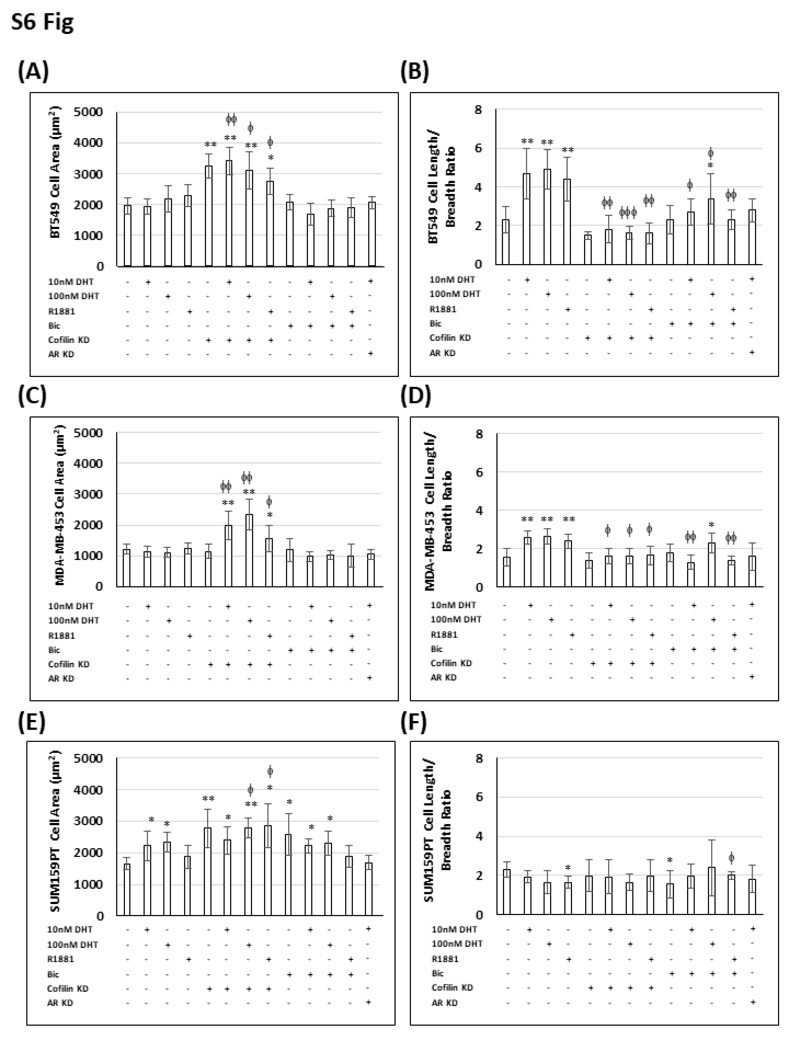

Supplement: S6 Fig — Control and treated TNBC cells were stained with fluorescent-conjugated phalloidin and cell area, length (the span of the longest chord through the cell), and breadth (the caliper width of the cell perpendicular to the longest chord) were measured by Metamorph software (Molecular Devices). (A) BT549 cell area (μm2). (B) BT549 length/breadth ratio. (C) MDA-MB-453 cell area (μm2). (D) MDA-MB-453 length/breadth ratio. (E) SUM159PT cell area (μm2). (F) SUM159PT length/breadth ratio. Data are expressed as mean ± SEM, n ≥ 100 cells in each experiment, three independent experiments. * p <0.05, ** p <0.01 versus control, ϕ p <0.05, ϕϕ p <0.01, ϕϕϕ p <0.001 versus androgen-treated cells. (TIF) [file pone.0279746.s008.tif]

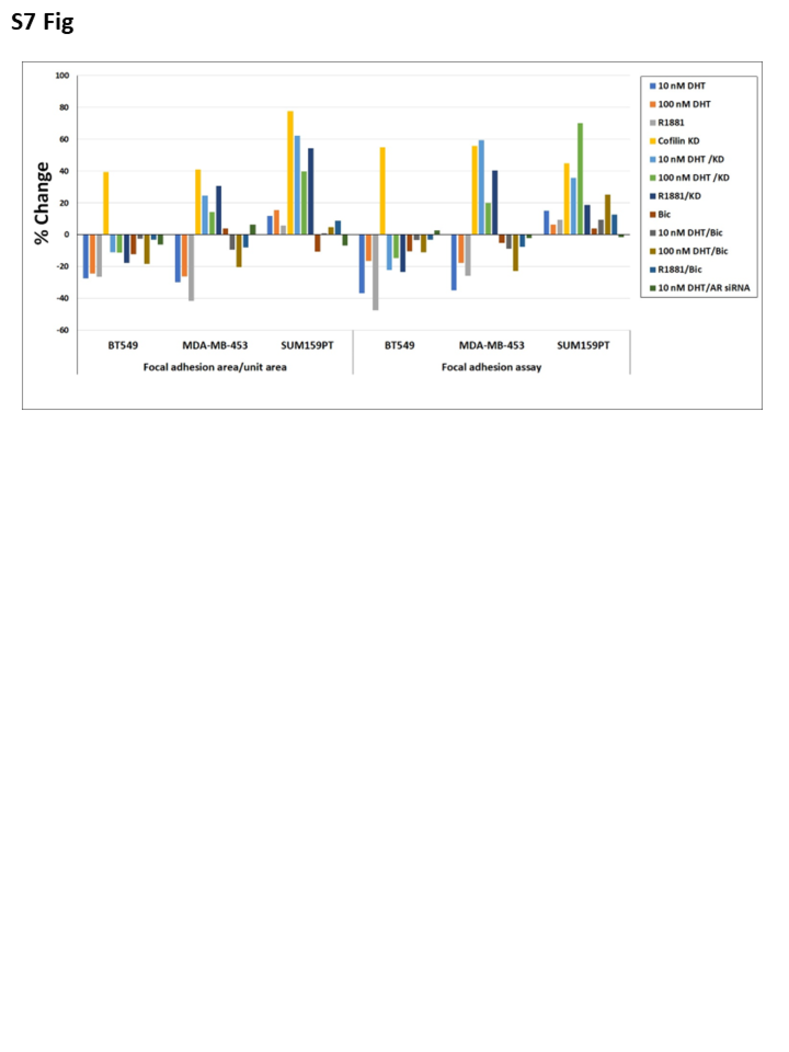

Supplement: S7 Fig — (TIF) [file pone.0279746.s009.tif]

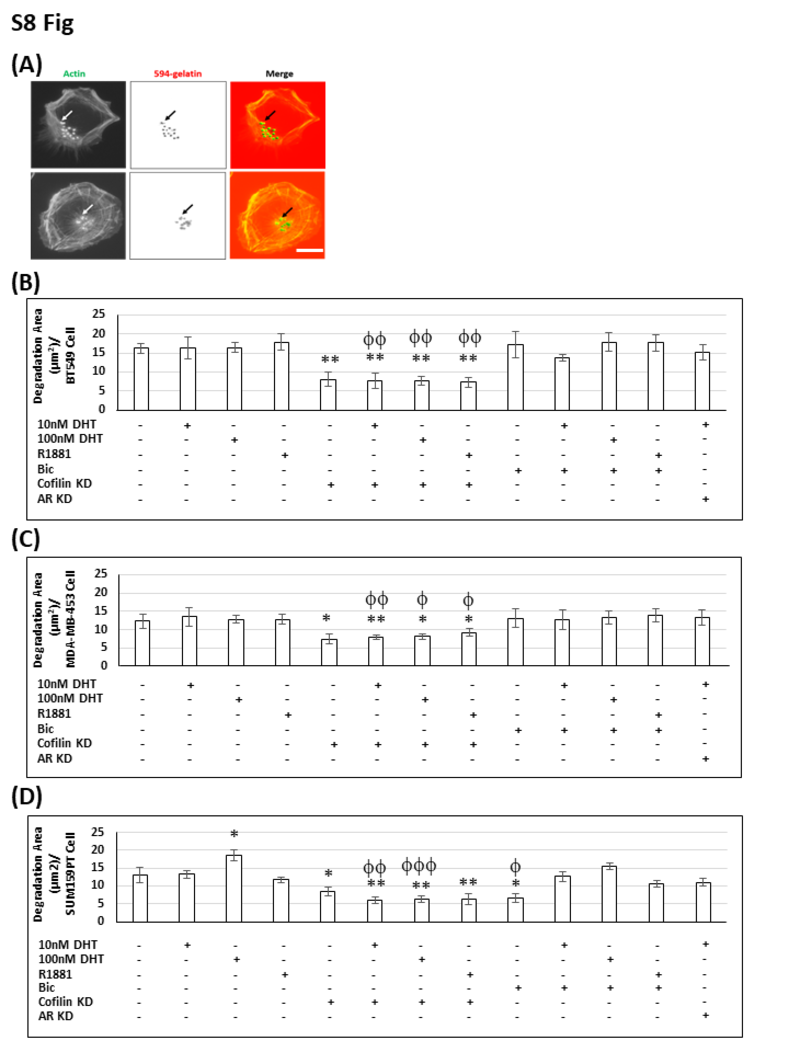

Supplement: S8 Fig — (A) Cells were cultured on fluorescent gelatin-coated cover slips and stained with fluorescent-phalloidin to visualize invadopodia (arrows). Scale bar: 10 μm. The degradation area of gelatin (μm2) was quantified in Metamorph and divided by number of cells in the same field and expressed as degradation area (μm2)/cell in (B) BT549 cells, (C) MDA-MB-453 cells, and (D) SUM159PT cells. n ≥ 25 cells in each experiment, three independent experiments. * p <0.05, ** p < 0.01 versus control, ϕ p < 0.05, ϕϕ p <0.01, ϕϕϕ p <0.001 versus androgen-treated cells. (TIF) [file pone.0279746.s010.tif]

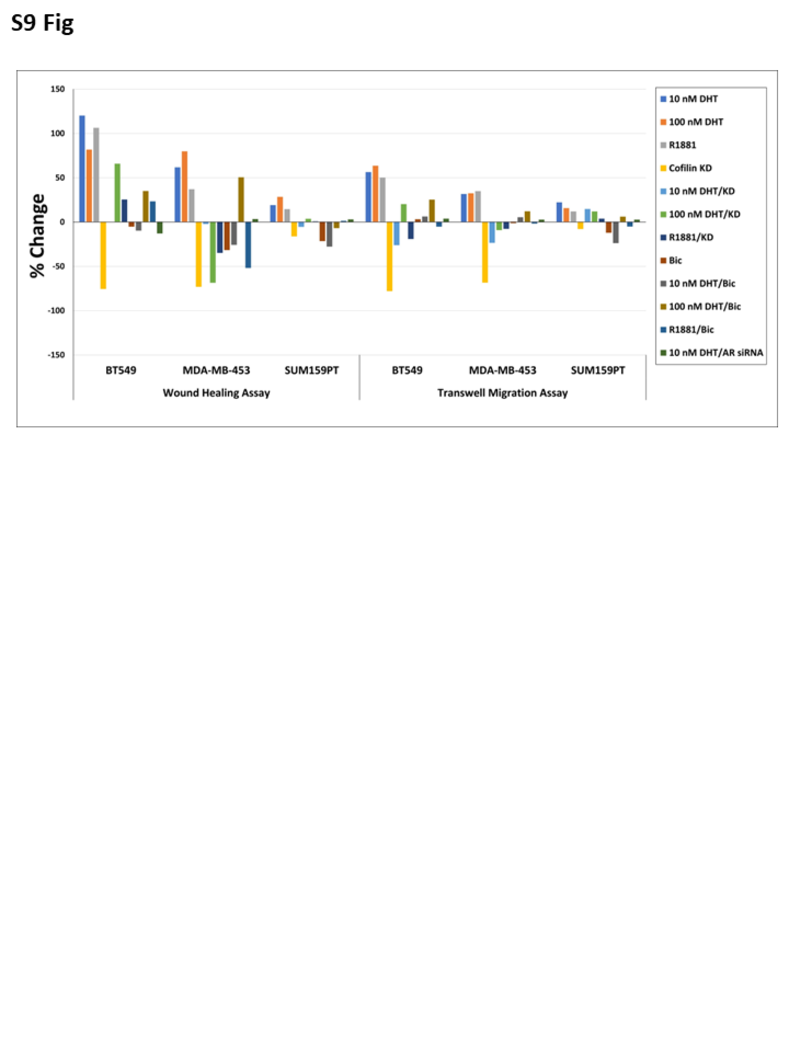

Supplement: S9 Fig — (TIF) [file pone.0279746.s011.tif]

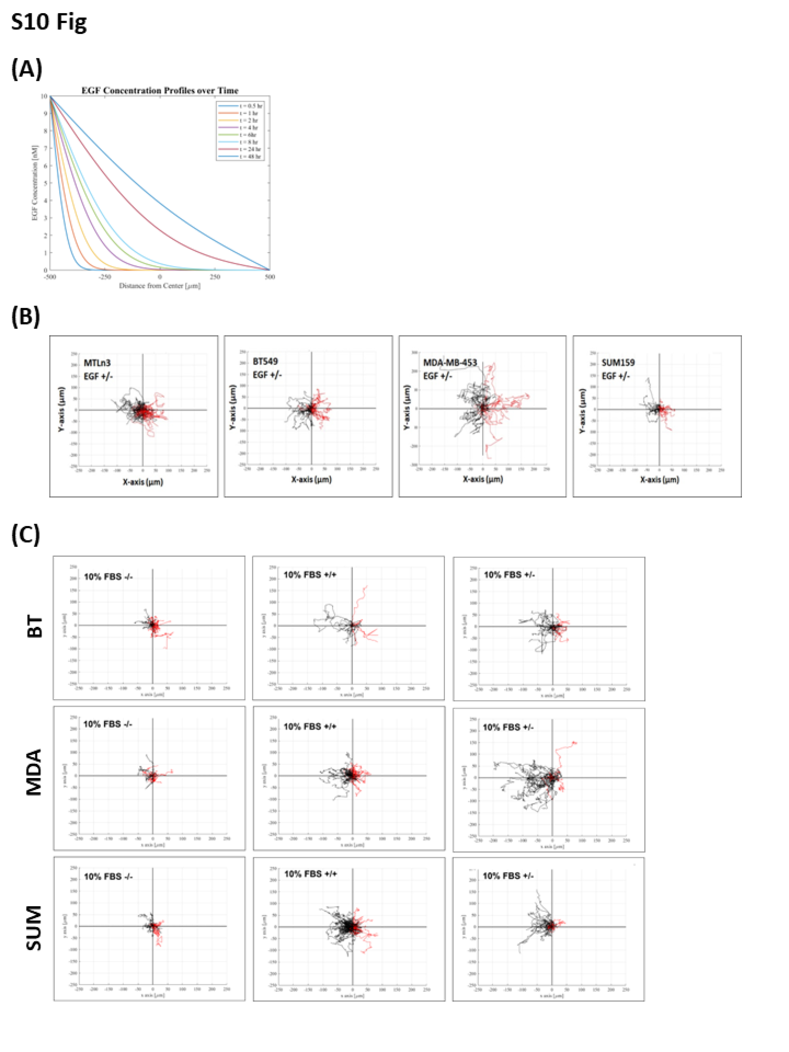

Supplement: S10 Fig — (A) EGF concentration profiles in the central chemotaxis chamber over time. For an initial concentration of 10 nM EGF in the left reservoir, it takes approximately 6 hr for the EGF diffusion front to cross the midpoint of the central chamber. The EGF concentration profile becomes nearly linear by 48 hr. (B) Control untreated cells were seeded on collagen IV-treated chemotaxis μ-slide with 10 nM EGF in the left reservoir and chemoattractant-free media in the right reservoir (EGF +/-). (C) Control and treated cells were seeded on collagen IV-coated chemotaxis μ-slide. 10% FBS -/-: negative/negative control cells with no chemoattractant in either reservoir, 10% FBS +/+: positive/positive control cells with 10% FBS in both reservoirs, 10% FBS +/-: positive/negative control or treated cells with 10% FBS in the left reservoir. Cell tracking was performed in MATLAB using the image processing software CellTracker. Cell trajectories were then imported into the ImageJ software plugin "Chemotaxis and Migration Tool" and extrapolated to (x,y) = (0,0) at time 6 hr, where the x-axis is parallel to the chemoattractant gradient and the y-axis is perpendicular to the gradient. On average 30–60 cells were tracked per experiment, and each experiment was repeated three times. (TIF) [file pone.0279746.s012.tif]
